# Effects of drought stress and water recovery on physiological responses and gene expression in maize seedlings

**DOI:** 10.1186/s12870-018-1281-x

**Published:** 2018-04-23

**Authors:** Xiangbo Zhang, Lei Lei, Jinsheng Lai, Haiming Zhao, Weibin Song

**Affiliations:** State Key Laboratory of Agrobiotechnology/National Maize Improvement Center of China/Key Laboratory of Crop Heterosis and Utilization of the Ministry of Education/Beijing Key Laboratory of Crop Genetic Improvement, China Agricultural University, No. 2, Yuanmingyuan West Road, Haidian District, Beijing, 100193 China

**Keywords:** *Zea mays*, Seedling, Drought stress, Water recovery, Photosynthetic efficiency, Transcription factor

## Abstract

**Background:**

Drought is one of the major factors limiting global maize production. Exposure to long-term drought conditions inhibits growth and leads to yield losses. Although several drought-responsive genes have been identified and functionally analyzed, the mechanisms underlying responses to drought and water recovery treatments have not been fully elucidated. To characterize how maize seedling respond to drought stress at the transcriptional level, we analyzed physiological responses and differentially expressed genes (DEGs) in the inbred line B73 under water deficit and recovery conditions.

**Results:**

The data for relative leaf water content, leaf size, and photosynthesis-related parameters indicated that drought stress significantly repressed maize seedling growth. Further RNA sequencing analysis revealed that 6107 DEGs were responsive to drought stress and water recovery, with more down-regulated than up-regulated genes. Among the DEGs, the photosynthesis- and hormone-related genes were enriched in responses to drought stress and re-watering. Additionally, transcription factor genes from 37 families were differentially expressed among the three analyzed time-points. Gene ontology enrichment analyses of the DEGs indicated that 50 GO terms, including those related to photosynthesis, carbohydrate metabolism, oxidoreductase activities, nutrient metabolism and other drought-responsive pathways, were over-represented in the drought-treated seedlings. The content of gibberellin in drought treatment seedlings was decreased compared to that of control seedlings, while abscisic acid showed accumulated in the drought treated plants. The deep analysis of DEGs related to cell wall development indicated that these genes were prone to be down-regulated at drought treatment stage.

**Conclusions:**

Many genes that are differentially expressed in responses to drought stress and water recovery conditions affect photosynthetic systems and hormone biosynthesis. The identified DEGs, especially those encoding transcription factors, represent potential targets for developing drought-tolerant maize lines.

**Electronic supplementary material:**

The online version of this article (10.1186/s12870-018-1281-x) contains supplementary material, which is available to authorized users.

## Background

Like all other crops, maize plants grown under natural conditions are exposed to various abiotic stresses throughout their life cycle [1–3]. Water deficit stress is considered as one of the most important environmental factors that adversely affect maize production [2, 4, 5]. A lack of water decreases the seedling survival rate and increases the post-pollination embryo abortion rate, ultimately leading to decreased yield [1, 6, 7]. In China, more than 60% of the agricultural land devoted to corn production has encountered long-term or seasonal drought conditions, which may reduce yields by as much as 30% [8]. To ensure high survival rates and production under drought conditions, maize plants rely on several strategies, including drought avoidance, escape and tolerance [9–11]. Consequently, several biological processes are affected through changing global gene expression patterns [12, 13]. Thus, characterizing the physiological responses and differentially expressed genes (DEGs) is important for clarifying the complex physiological mechanisms underlying drought stress responses.

Extended periods of water deficit will result in smaller leaves, premature flowering and a longer anthesis–silking interval, ultimately decreasing yield potentials [14, 15]. Maize seedlings growing under water stress conditions exhibit several important physiological responses, including decreased cell turgor [7, 16], leaf rolling [17], inhibited CO_2_ exchange and decreased photosynthetic efficiency and chlorophyll contents [1, 18, 19]. The photosynthetic and gas exchange responses are the most sensitive to water deficits [20], and maintaining relatively high photosynthetic activity levels may enhance plant drought tolerance. Over-expression of heat shock transcription factor (TF) HSFA9 may increase water deficit tolerance by protecting the photosynthetic complex in seedlings of tobacco [21]. A recent study revealed that over-expression of the photosystem-associated maize *psbA* gene in tobacco enhanced drought resistance via increasing photosynthetic capabilities [22]. However, a high-throughput identification of photosystem-associated genes and a clarification of the relationship between these genes and drought stress responses are required.

An increasing number of genes responsive to water stress have been isolated and functionally characterized, including genes related to photosynthesis, and metabolism-regulating synthetic enzymes [1, 2, 22–24]. Plants over-expressing *ZmNF-YB2*, which encodes the nuclear factor-Y subunit B2, were observed to exhibit increased tolerance to water deficit stress based on most drought-related parameters, including leaf rolling and seedling leaf temperature, as well as a field trial [2]. It was recently reported that the ectopic expression of the TF-encoding *ZmNAC111* led to enhanced drought tolerance under water deficit stress in 2-week-old seedlings [1]. Additionally, the transgenic maize plants exhibited higher water-use efficiency under drought conditions, while there were no phenotypic differences between normally irrigated transgenic and wild-type plants [1]. However, additional research is required to determine if there are any yield differences between the transgenic and wild-type plants grown under field conditions [1]. The expression of *ZmGOLS2*, which encodes galactinol synthase 2, is induced by various abiotic stresses [25, 26]. Over-expression of *ZmGOLS2* significantly increases galactinol and raffinose contents and results in enhanced drought tolerance in *Arabidopsis thaliana* plants. Interestingly, expression of *ZmGOLS2* is regulated by ZmDREB2A TF, which reportedly affects maize drought tolerance [18, 27]. Trehalose influences several biological processes in rice seedlings [28, 29]. Furthermore, over-expression of a rice trehalose-6-phosphate phosphatase gene under the control of a flower-specific promoter leads to the accumulation of sucrose in the ear. Field trials revealed that the transgenic maize grain yield was significantly higher than that of non-transgenic controls under mild and severe drought stress conditions [4]. These findings suggest that the up- or down-regulated expression of genes encoding TFs or metabolic factors can increase maize drought tolerance during the seedling and reproductive stages. Additionally, identifying DEGs responsive to drought stress using RNA sequencing (RNA-seq) technology may provide useful information for elucidating the mechanisms mediating drought stress responses [30].

RNA sequencing is a classical technique that has been used to identify drought-responsive pathways or genes that are active during the seedling stage under various abiotic stress conditions. Min et al. [13] analyzed the biological responses of maize seedlings to drought stress at three time-points using RNA-seq, with the pre-stress values serving as controls. Meanwhile, Shan et al. [31] evaluated the crosstalk between gene expression and metabolic activities in responses to cold, drought and salt stresses. They identified many stress-responsive genes, and they also analyzed some genes based on the RNA-seq data. Opitz et al. [32] used RNA-seq to investigate the gene expression differences in the roots of maize plants exposed to drought conditions for 6 or 24 h. Although these studies identified many drought-responsive genes and pathways using various plant materials, the molecular responses to a water recovery period following a long-term exposure to drought conditions have not been characterized. To clarify the molecular responses to drought stress and water recovery treatments during the seedling stage, we compared the gene expression levels of drought-stressed and control maize seedling. In the present study, reference line B73 was used to investigate the physiological responses and global gene expression patterns induced by 3-day drought, 6-day drought, and 1-day water recovery treatments. The gene expression patterns of seedlings after a drought treatment and water recovery period were identified based on RNA-seq data. The identified drought-responsive genes may be useful for analyzing the mechanisms regulating maize seedling responses to drought stress and re-watering.

## Methods

### Plant growth and drought treatments

The seeds of inbred line B73 were preserved in CAU National Maize Improvement Center in our lab. The seeds that were used for drought treatments and RNA-seq analysis were sterilized and germinated in our laboratory. Germinated seeds were planted in pots (diameter: 10 cm; 10 seedlings per pot), which were transferred to a greenhouse and treated as described in our previous study [11]. Seedlings were grown at 25 ± 2 °C and 60–70% humidity, with an 18-h photoperiod provided by natural sunlight. Three controls were collected at each time-point to eliminate the effects of growth and development processes. Eighteen pots were divided into control and drought-treatment groups (i.e., nine pots each) for a total of three replicates, with three pots each. Two replicates were randomly selected for the subsequent phenotyping and RNA sequencing. All plants were watered daily until the three-leaf stage to ensure seedlings were able to grow in soil with sufficient water (i.e., 11 days from germination to the three-leaf stage). We irrigated the nine control pots every day at the same time to keep soil wet. Water was withheld from the other nine pots (i.e., natural drought stress) for 3 days until the leaf rolling phenotype was observed. For a more severe drought treatment, water was withheld for another 3 days, which resulted in severe leaf rolling. The 6 day drought-treated samples were then re-watered after 24 h. For each time-point (i.e., 3-day drought, 6-day drought, and water recovery period), seedling phenotypes were assessed before plants were harvested. Drought-related characteristics, including leaf relative water content (RWC), leaf length, and photosynthesis-related parameters, were measured before sampling. Two replicates of harvested seedling shoots (i.e., aerial parts) were divided into two parts. The first part was used to construct the RNA-seq library, while the second part was used for a quantitative reverse transcription polymerase chain reaction (qRT-PCR) analysis. Yellow and gray leaf tips were removed prior to freezing harvested samples in liquid nitrogen.

### Evaluation of relative water content

To evaluate the effects of drought treatments, we measured the seedling RWC. Briefly, paper bags were first baked at 65 °C for 3 days until reaching a constant weight. Fresh leaves were weighed (WF) and then soaked in distilled water for 24 h. The leaves were weighed again to obtain the saturated weight (WFT), after which they were fixed at 105 °C for 30 min. The leaves were then placed in the dried paper bags and incubated at 80 °C for 3 days. Three independent samples were used to determine the constant dry weight (WD). The RWC was calculated based on the following formula: RWC = (WF − WD) / (WFT − WD) × 100%. The Student’s *t*-test was used to detect significant differences (*P* < 0.01) between the data for the drought-treated and control samples. Data from two biological replicates (four plants per replicate) were analyzed, and are presented in the figures as the mean of two replicates ± standard deviation (SD).

### Measurement of leaf length

The length of the second seedling leaf for all samples was measured after the 3-day drought, 6-day drought, and 1-day water recovery treatments. A ruler was used to measure the length from the leaf tip to the sheath for 20~ 25 plants at each time-point. The leaf length data underwent a one-way analysis of variance using Microsoft Excel software. The Student’s *t*-test was used to detect significant differences (*P* < 0.01) between the data for the drought-treated and control samples. Data from two biological replicates (four plants per replicate) were analyzed, and are presented in the figures as the mean of two replicates ± SD.

### Gas exchange rate and chlorophyll fluorescence measurement

The gas exchange rate and chlorophyll fluorescence following different drought treatments were measured using the LI-6400 portable photosynthesis system (LI-COR Company, Lincoln, NE, USA) according to the manufacturer instructions with some modifications. First, seedlings in pots were kept in one large dark box for 40 min to determine the minimum (Fo) and maximum (Fm) fluorescences. The Fo was recorded under the lowest modulated light conditions, while the Fm and variable chlorophyll fluorescence (Fv) were assessed after an exposure to saturating white light (6000 μmol m^− 2^ s^− 1^) for 0.8 s. Steady-state fluorescence (Fs) was measured by exposing plants to white light (500 μmol m^− 2^ s^− 1^) until the leaf photosynthetic activity reached a steady-state. A second maximum fluorescence (Fm′) was recorded following another exposure to saturating white light (6000 μmol m^− 2^ s^− 1^) for 0.8 s. The highest quantum efficiency of photosystem II (PSII) was calculated using the following formula: Fv/Fm = (Fm − Fo)/Fm , while the actual quantum yield of PSII electron transport was determined as follows: ΦPSII = (Fm^′^ − Fs)/Fm^′^. The measurements involved the third leaf of each plant. Two biological replicates were analyzed, with three plants per replicate.

### Measurement of chlorophyll contents

The drought-induced changes to chlorophyll contents were assessed using a SPAD-502 (Soil and Plant Analyzer Development) portable chlorophyll meter (Konica Minolta Inc., Tokyo, Japan). The third fully expanded leaf (from the top) of each seedling was analyzed after the 3-day drought, 6-day drought, and 1-day water recovery treatments. Each leaf was analyzed three times at different sites. The chlorophyll content of each leaf was based on the average of three readings. The measurement was completed using two biological replicates, with four plants per replicate. The average of all readings was used for the following data analysis. Data are presented in figures as the mean of two replicates ± SD.

### Total RNA extraction, qRT-PCR, and RNA sequencing

Total RNA was extracted from B73 seedling shoots (i.e., aerial parts) using Trizol reagent (Invitrogen). For the qRT-PCR analysis, the extracted total RNA was treated with RQ1 RNase-free DNase (Promega), after which first-strand cDNA was amplified using M-MLV Reverse Transcriptase. The qRT-PCR was completed using the ABI 7500 Real-Time PCR System (Applied Biosystems, USA) and SYBR Premix (Thermo Scientific, USA). A more thorough description of the qRT-PCR procedure is provided in one of our previous publications [11], and the primers used to amplify the nine genes were designed with the Premier 5 (v5.0) program (see Additional file 1). Two independent experiments were completed, each with three technical replicates. The results of a representative experiment are provided, with data presented as the mean ± SD (*n* = 3). The extracted total RNA was also used to prepare RNA-seq libraries according to the Illumina Standard mRNA-seq Library Preparation kit (Illumina). The RNA-seq was completed using the Illumina HiSeq 2000 system as previously described [11]. The RNA-seq experiment (including the library construction) was completed with two biological replicates.

### Identification of differentially expressed genes

The 125-bp paired-end reads generated by the Illumina HiSeq 2000 system were aligned with the B73 reference genome (v2) using TopHat (v2.0.6) [33], with default settings for all parameters. The unique mapped reads were used in the following analyses. The default parameters of the Cuffdiff (v2.2.1) program were used to analyze gene expression levels in terms of fragments per kilobase per million mapped reads (FPKM) and to identify DEGs [34]. The genes with an absolute log_2_ fold change value (treated/control) ≥ 1 (adjusted *P* ≤ 0.05 [32]) were considered as DEGs. The RNA-seq data were deposited in the NCBI database (Accession number is: SRP101911; https://www.ncbi.nlm.nih.gov).

### Gene ontology enrichment, MapMan annotation and gene clustering

We used the default settings of the agriGO online tool (http://bioinfo.cau.edu.cn/agriGO/) to analyze the functional enrichment of all DEGs. Significant GO terms (q ≤ 0.05) were selected. Different metabolic pathways associated with the DEGs were identified with the MapMan program [35]. The up- and down-regulated genes are indicated in red and blue, respectively. The MapMan program is a user-driven tool that displays genomic data sets on diagrams of metabolic pathways and other biological processes. For the cluster classification, the DEGs were grouped into 10 clusters with the K-means algorithm of the MultiExperiment Viewer program (v4.9.0) based on the log_2_ fold change values (treated/control).

### Prediction of photosynthesis-related genes using BLASTP

The protein sequences encoded by the DEGs associated with the light-harvesting complex (LHC), PSII, and photosystem I (PSI) were used as queries in a BLASTP search of the Nr database to obtain a full annotation. An E-value < 0.01 was selected as the cutoff.

### Measurements of the contents of GA, ABA and SA

Control and drought treatment seedlings of 3d, 6d and re-watered were used to measure the contents of GA, ABA and SA, respectively. Three replicates were prepared at each time points. We measured their contents according to the instructions of standard hormonal kit (ELISA): MM-012601 for GA, MM-013801 for ABA, and MM3372201 for SA (products of Jiangsu JingMei Bio.Company).

## Results

### Physiological responses to drought stress and water recovery

**Fig. 1:**
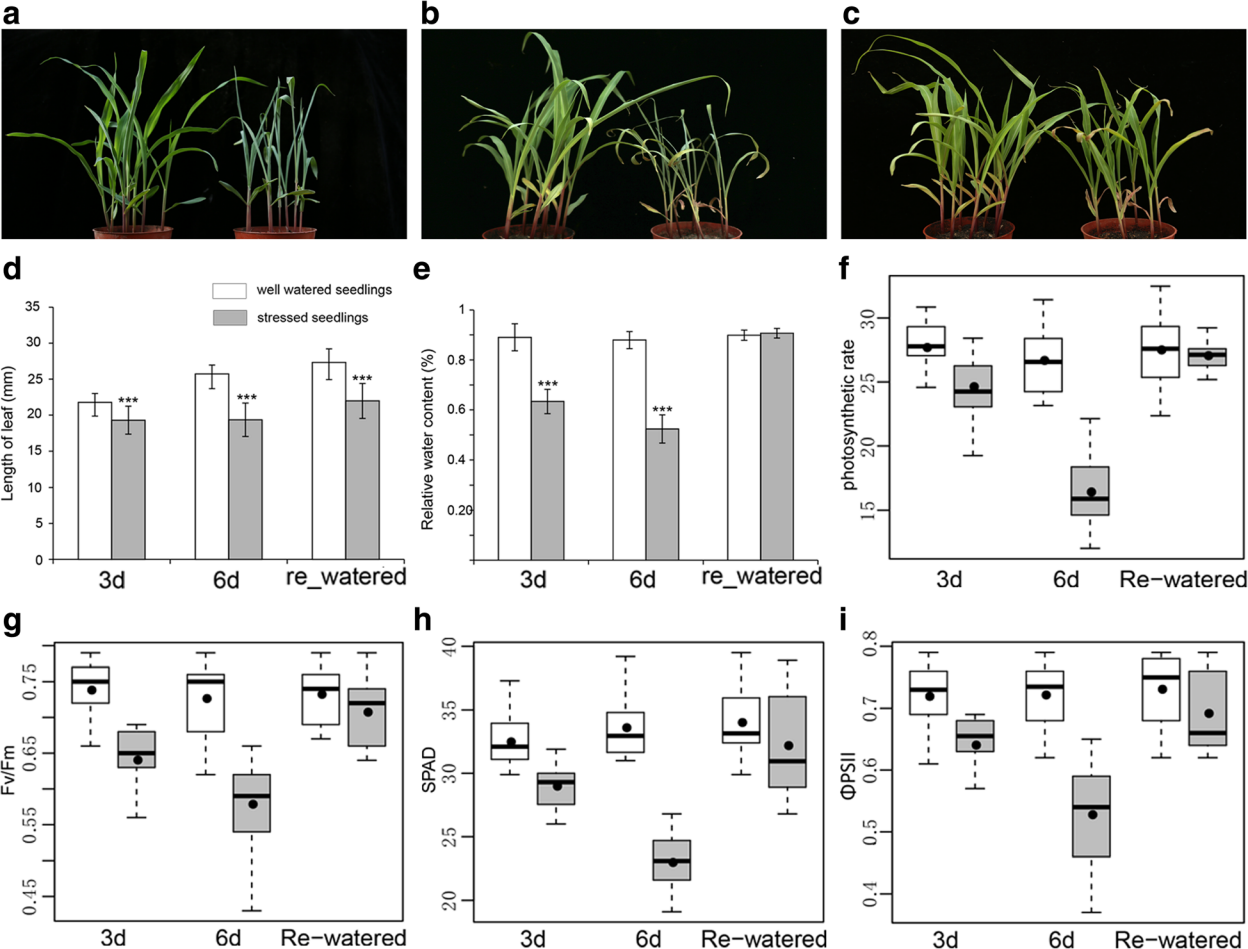
Physiological responses of seedling leaves affected by drought treatments and the water recovery period. Phenotypic responses of B73 seedlings to drought stress (DS) and water recovery treatments at different time points (**a**: 3-day drought; **b**: 6-day drought; **c**: 1-day water recovery). The pots on the right and left correspond to the drought-treated and well-watered control plants, respectively. The relative water content and leaf length were measured using seedling leaves after a 3-day (3d) or 6-day drought treatment (6d) and a 1-day water recovery period (re-watered, **d** and **e**). The values in **d** and **e** are presented as the mean ± standard deviation of three biological replicates, with each replicate consisting of three plants. The asterisks indicate significant differences (*P* < 0.001) according to the Student’s *t*-test. The leaf length (**d**), relative water content (**e**), photosynthetic rate (**f**), Fv/fm (**g**), SPAD (**h**), and ΦPSII (**i**) values were recorded for drought-treated (grey) and control seedlings (white) at three time points. All measurements were completed with the third seedling leaf

To investigate the physiological responses of maize seedlings to water deficit and recovery, the phenotypic traits, including RWC and leaf length, were evaluated at the following three time-points: 3 and 6 days after initiating the drought treatment and after a 1-day water recovery period (Fig. 1a-c). The RWCs of drought-treated leaves decreased to 62.7% and 49.8% after 3 and 6 days, respectively (Fig. 1d). Meanwhile, the RWCs of the drought-treated and control seedlings were similar following the water recovery period. Additionally, the drought-treated leaves were significantly shorter than the control leaves after the 3-day drought, 6-day drought, and 1-day water recovery treatments (Fig. 1e). Then we calculated elongation rate of leaf between control and drought treatment seedlings (see Additional file 2: Figure S1). In the drought treatment stage (3d~6d), the elongation rate of the drought treatment seedlings were lower than control seedlings, which best matched the short leaf and lower photosynthetic rate in the drought seedlings. But in the re-watered stage (6d~re-watered), the rate of drought treatment seedlings were higher than control samples. This might be explained by the high water absorption of the re-watered seedlings. The other analyzed phenotypic traits were also significantly affected by water deficit stress. For example, at 3-day and 6-day drought treatment, the leaves were wilted and obviously rolled. In contrast, the leaves of seedlings that were normally watered (i.e., controls) no changes after 3 and 6 days. After re-watering for 24 h, the leaves of all drought-treated plants recovered and were more similar to the controls compared to drought stressed plants (Fig. 1a–c). However, the re-watered drought-treated seedlings remained smaller than the controls and some leaf tips were gray or yellow (Fig. 1e). These results indicated that seedling growth was inhibited by drought conditions.

Photosynthetic systems are susceptible to damage during responses to water deficit stress [22]. To determine the extent of the drought-induced damages to these systems, photosynthesis-related parameters of both stressed and re-watered seedlings, including the photosynthetic rate, chlorophyll content and luminous energy, were measured with the LI-6400 portable photosynthesis system and the SPAD-502 portable chlorophyll meter. The photosynthetic rate decreased in response to water deficit stress, with the lowest value observed for the 6-day drought treatment, but then recovered after plants were re-watered (Fig. 1f). Changes to the three other analyzed parameters [i.e., Fv/fm, chlorophyll content (SPAD value) and ΦPSII during the drought and water recovery treatments were similar to the photosynthetic rate changes (Fig. 1g–i). The results demonstrated that drought inhibits maize photosynthesis. The inhibition was enhanced by the aggravation drought stress and weaken by relief of the stress.

### Global gene expression profiles induced by drought stress and water recovery

To clarify the molecular mechanisms regulating maize responses to drought stress and water recovery treatments, the shoots sampled from drought-treated, re-watered, and control seedlings were used to investigate gene expression patterns by RNA-seq. We obtained over 132 million unique reads from an Illumina sequencing system at each time-point, with an average of 84.33% of the reads being mapped to the B73 reference genome covering approximately 20,000 predicted genes (FPKM ≥1) (see Additional file 3 and Additional file 2: Figure S2A). By PCA analysis, control and drought treated seedlings in the 3d and 6d time points were clustered apart (see Additional file 2: Figure S3). At the re-watered stage. The control and drought treated seedlings were clustered together. The similarity in the results for two biological replicates confirmed the data were reproducible (see Additional file 2: Figure S4).

**Fig. 2:**
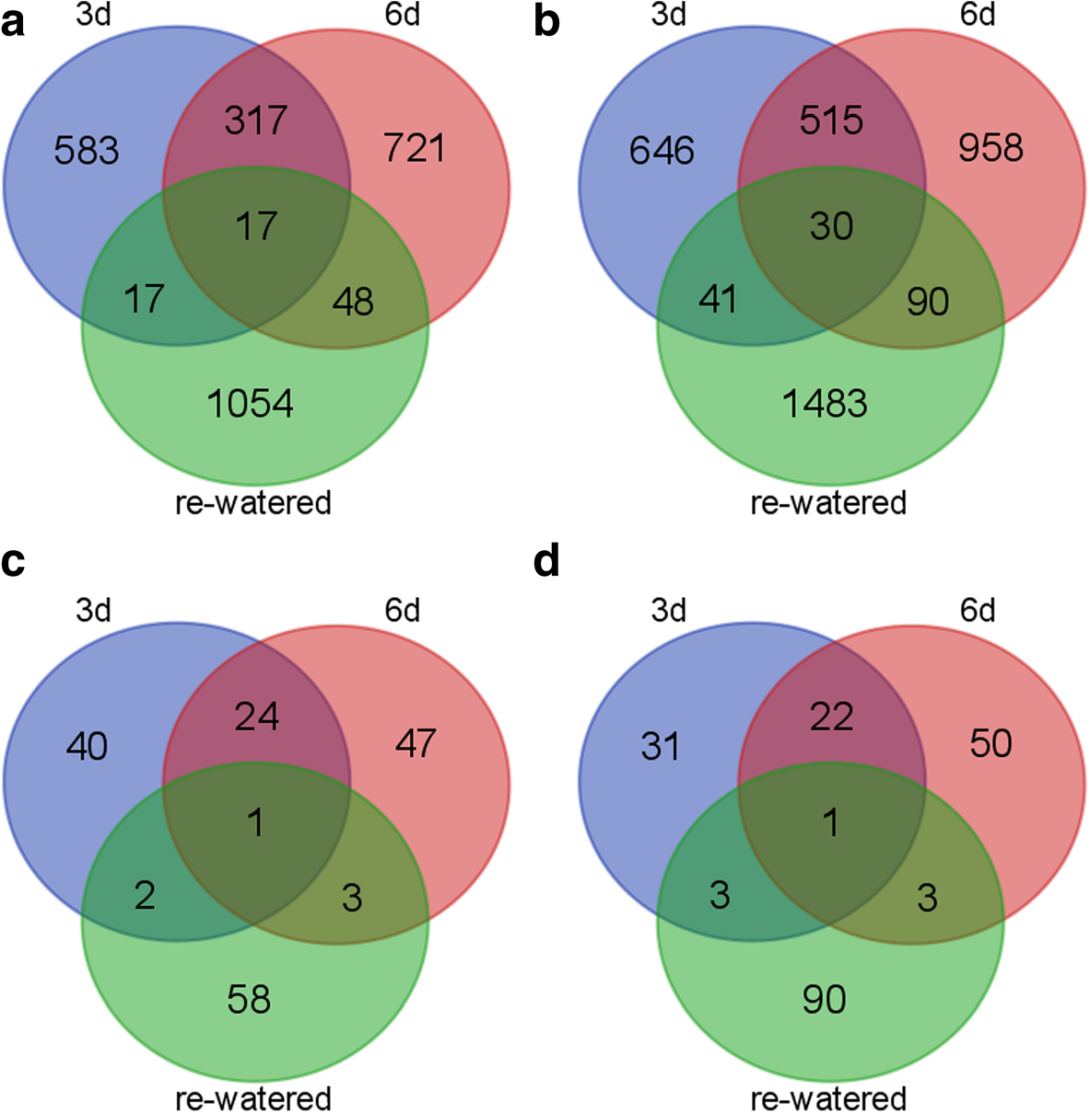
Global gene expression profiles and the identification of DEGs in response to the treatments. Venn diagrams illustrate the overlap between the differentially expressed genes identified following the 3-day drought, 6-day drought, and 1-day water recovery treatments. Up-regulated genes (**a**). Down-regulated genes (**b**). Up-regulated TF genes (**c**). Down-regulated TF genes (**d**)

We detected 6107 genes that were differentially expressed among the three time-points (see Additional file 4 and Additional file 5), including 2757 and 3763 up- and down-regulated genes, respectively (Fig. 2a and b). The fact that there were more down-regulated than up-regulated genes under drought conditions implies that drought stress tends to inhibit gene expression globally. Further analyses revealed that the gene expression levels were similar among the treated and control seedlings after the 3-day drought, 6-day drought and water recovery period (see Additional file 2: Figure S2B). We next compared the DEGs detected for the two drought treatment time-points and the water recovery period. Among the up-regulated genes, 334 DEGs were detected at both drought treatment time-points (Fig. 2a). In contrast, only 34 DEGs were common to the 3-day drought treatment time-point and after the water recovery period. Only 65 common DEGs were detected after the 6-day drought treatment and following the water recovery period (Fig. 2a). Similarly, 545 down-regulated DEGs were identified at both drought treatment time-points. This was far more than the number of DEGs common to the 3-day drought-treated and re-watered samples (i.e., 71) as well as the 6-day drought-treated and re-watered samples (i.e., 120) (Fig. 2b). These differences suggest that the mechanism mediating the drought response at both time-points differed from that regulating the response to being re-watered.

Drought will stress every stage during maize growth and development, but the comparison between the effects of drought on maize different physiological stages was less. To find coincidence between maize seedlings with the silk and ovary under drought treatment, we compared our data with Oury’s data [36, 37], which included microarray chip data (see Additional file 6). When compared to genes involved in expansive growth in silk, which were the cause of ovary abortion, we detected three genes which showed differential expression (two genes in 6d and one genes in re-watered stage) in both our data and that of Oury’s. On the other hand, when compared to DEGs involved in Carbon metabolism and expansive growth detected in ovaries, 37 and 55 DEGs were detected. Interestingly, more DEG were detected in 6d than 3d. These overlapped DEGs might have the potential for breeding as markers.

### Transcription factor gene expression induced by drought stress and water recovery

Many TFs, such as ZmDREB2A [18] and ZmNAC111 [1], are important for drought stress responses in maize seedlings. To further investigate the responses of TFs, we analyzed TF gene expression on a genome-wide scale at each time-point. Overall, the TF genes were expressed at lower levels than the non-TF genes (see Additional file 2: Figure S2B). Further analyses revealed that the TF gene expression levels at the 3-day drought treatment time-point and after the water recovery period were similar to the control levels (see Additional file 2: Figure S2B). However, the TF gene expression levels in the 6-day drought-treated samples were higher than in the corresponding control samples (see Additional file 2: Figure S2B). Among these expressed TF genes, 359 were differentially expressed between the drought and water recovery conditions (see Additional file 7 and Additional file 8). Unlike the total number of DEGs, there was a similar number of up- and down-regulated TF genes (Fig. 2c and d). Of the differentially expressed TF genes, those encoding NAC, MYB-related, bZIP, bHLH, and ERF TFs were over-represented (see Additional file 7 and Additional file 8; Additional file 2: Figure S5). Further analysis revealed that only one up-regulated TF gene, belonging to the NAC family, was common to all three time-points (Fig. 2c; see Additional file 7). Similar results were observed for the down-regulated TF genes (Fig. 2d; see Additional file 8).

The *ZmNAC111* TF influences drought responses by regulating the expression of several downstream genes [1]. We attempted to identify more of these candidate downstream genes of *ZmNAC111* using our data and the publicly available qTeller data (http://www.qteller.com/). We detected the top 100 co-expressed genes with *ZmNAC111* using the two datasets, respectively. As a result, we overlapped the top 100 genes to get the putative targets. Consequently, six candidate genes were identified, including three (i.e., GRMZM2G102183: malate synthase; GRMZM2G32037: nonspecific lipid-transfer protein precursor; GRMZM2G340656: stachyose synthase precursor) related to nutrient metabolism. Of the remaining three genes, one (i.e., GRMZM2G481005) was associated with a salt pathway, and two (i.e., GRMZM2G004548 and GRMZM2G181362) encoded a zinc-binding protein and a lysine-ketoglutarate reductase (see Additional file 2: Figure S6).

### Gene ontology classifications for the three time-points

**Fig. 3:**
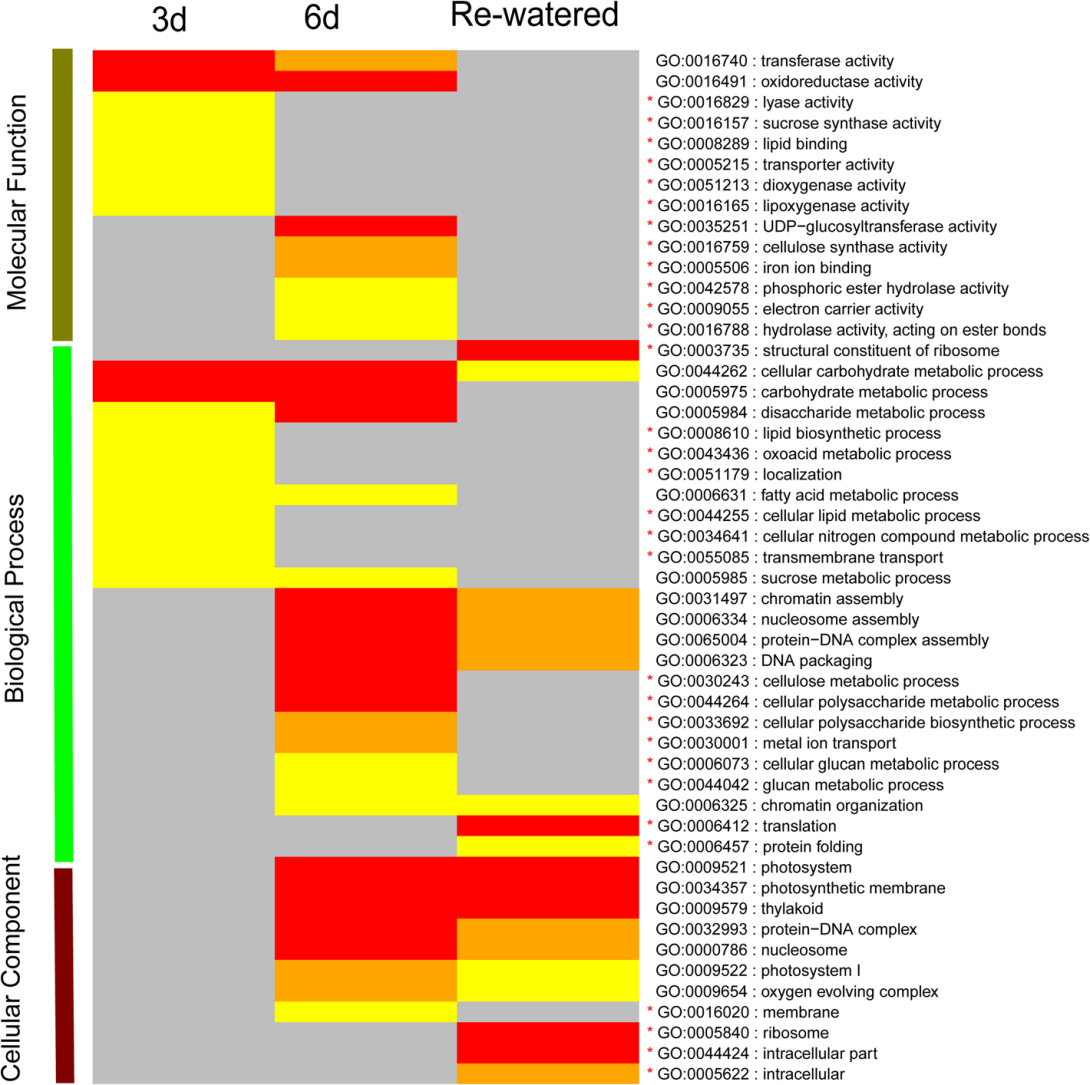
Enriched GO terms among the DEGs in response to water deficit and recovery. Different colors represent different significance levels [yellow: FDR < 0.05, orange: FDR < 0.01, red: FDR < 0.001, and gray: FDR > 0.05 (i.e., not significant)]

To compare the function enrichment of the up-regulated genes to that of down-regulated genes, a gene ontology (GO) enrichment analysis was performed based on AgriGO program (http://bioinfo.cau.edu.cn/agriGO/). The up- and down-regulated genes for the 3-day drought, 6-day drought and water recovery treatments were analyzed to identify significantly enriched GO terms, with different colored q-values used to visualize varying significance levels (Fig. 3; see Additional file 9). The common and unique over-represented GO terms at the three analyzed time-points are summarized in Fig. 3 and Additional file 9. Fifty significant GO terms were identified among all time-points, including seven enriched GO terms identified for both the 3-day and 6-day drought treatments (i.e., GO: 0016740, transferase activity; GO: 0016491, oxidoreductase activity; GO: 0044262, cellular carbohydrate metabolic process; GO: 0005975, carbohydrate metabolic process; GO: 0005984, disaccharide metabolic process; GO: 0005985, sucrose metabolic process; GO: 0006631, fatty acid metabolic process). Four of the seven over-represented GO terms were related to sugar metabolism. The cellular carbohydrate metabolic process (GO: 0044262) GO term was enriched at all three time-points (Fig. 3). Twelve GO terms that were enriched only at the 3-day drought treatment time-point were detected (indicated by red asterisks in Fig. 3). The GO terms associated with cell survival were identified after the water recovery period (i.e., GO: 0006457, protein folding; GO: 0044424, intracellular part; GO: 0005622, intracellular).

To further characterize the metabolic changes regulated by drought stress and water recovery, we visualized the metabolic responses to water deficit and recovery using the MapMan program [35] (see Additional file 2: Figure S7). There were 404 DEGs involved in six main metabolic activities related to the minor CHO, cell wall, lipids, starch and sucrose, TCA, as well as light reactions at the 3-day drought treatment time-point. Additionally, 544 metabolism-related DEGs were identified in the samples exposed to drought conditions for 6 days. Meanwhile, 400 DEGs related to metabolic activities were detected in the re-watered plants (see Additional file 10). Among the differentially regulated metabolic activities, those related to the light reaction were the most over-represented (see Additional file 2: Figure S7A–C). We also detected 47, 57, and 28 DEGs related to the sucrose synthesis and degradation pathways in the plants exposed to the 3-day drought, 6-day drought, and water recovery treatments, respectively (see Additional file 2: Figure S7D–F). These results suggest that sucrose synthesis and degradation as well as light reactions may play important roles in maize seedling responses to drought stress and water recovery.

### Cluster analysis of differentially expressed genes during the drought and water recovery treatments

**Fig. 4:**
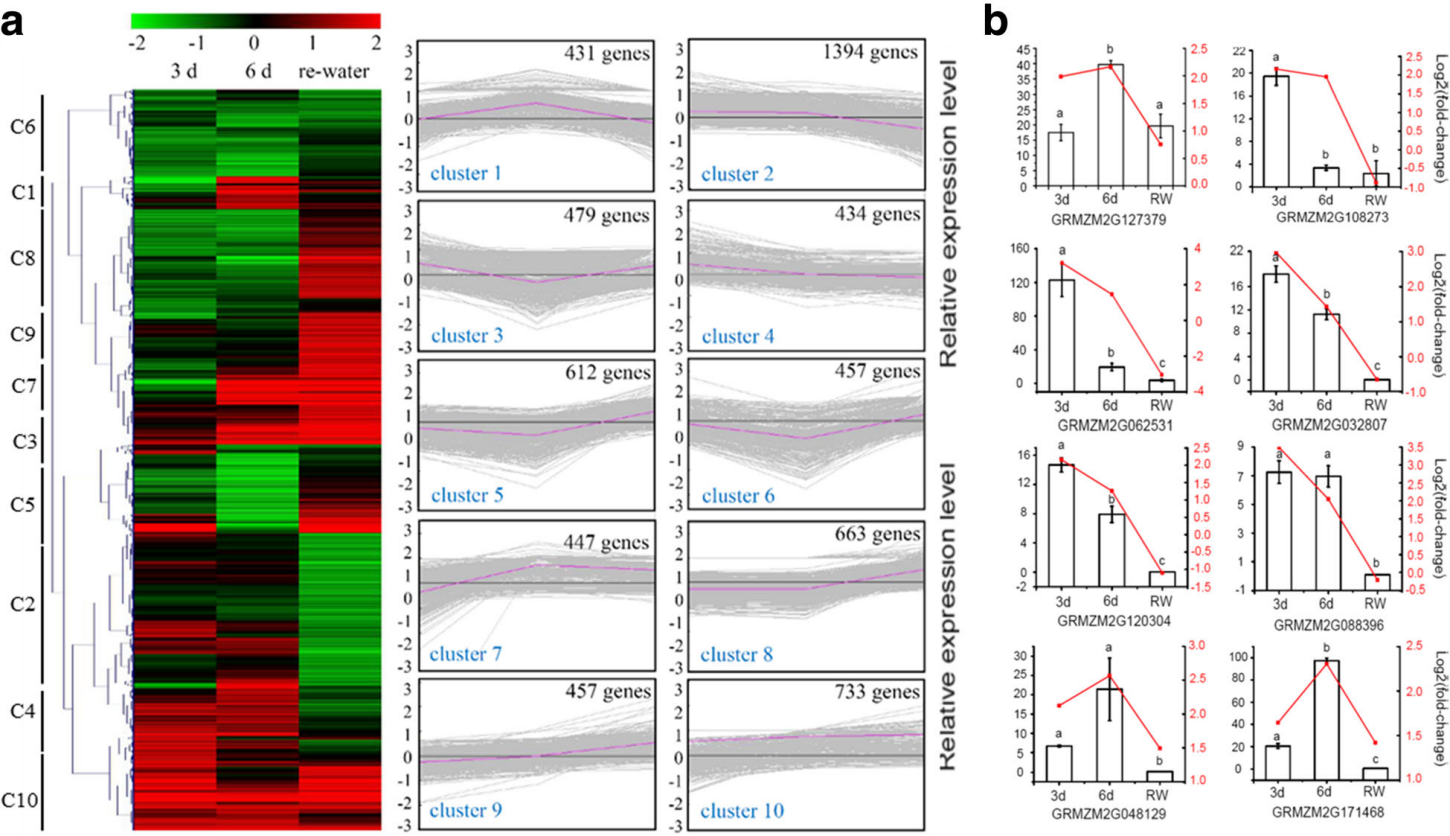
Cluster analysis of differentially expressed genes during drought treatments. Heat map illustrating the expression profiles of the differentially expressed genes at three time-points (i.e., 3-day drought, 6-day drought, and water recovery). The bars on the left side of represent the 10 different clusters, while the results of the cluster analysis of the gene expression profiles with the K-means algorithm are presented on the right side of (**a**). Randomly selected differentially expressed genes were analyzed by qRT-PCR. The relative gene expression levels based on the RNA-seq data are indicated by red lines. The letters a, b, and c indicate significant differences according to the Student’s *t*-test (*P* < 0.05) (**b**)

The FPKM values derived from the RNA-seq data were used to analyze the gene expression patterns among the three time-points. A heat map was generated based on all 6107 DEGs (*P* ≤ 0.05). To reflect the major trends and patterns, all DEGs were assigned to 10 clusters using a K-means algorithm (Fig. 4a). The Cluster 1 genes were most highly expressed after the 6-day drought treatment. The expression levels returned to normal during the subsequent water recovery period. Meanwhile, the expression levels of the Cluster 2 genes were up-regulated after the 3-day and 6-day drought treatments, and then down-regulated after plants were re-watered. The expression levels of the genes in Clusters 5, 6, and 8 were down-regulated by drought conditions, and then up-regulated when watering was resumed. In contrast, the Cluster 10 genes were up-regulated at all three time-points. These results indicate that the drought and water recovery treatments had variable effects on gene expression levels. To verify the results for the DEGs in the 10 clusters, the expression levels of eight randomly-selected drought-responsive genes were analyzed in a qRT-PCR experiment (Fig. 4b).

### Changes to the expression levels of photosynthesis- and hormone-related genes after drought and water recovery treatments

**Fig. 5:**
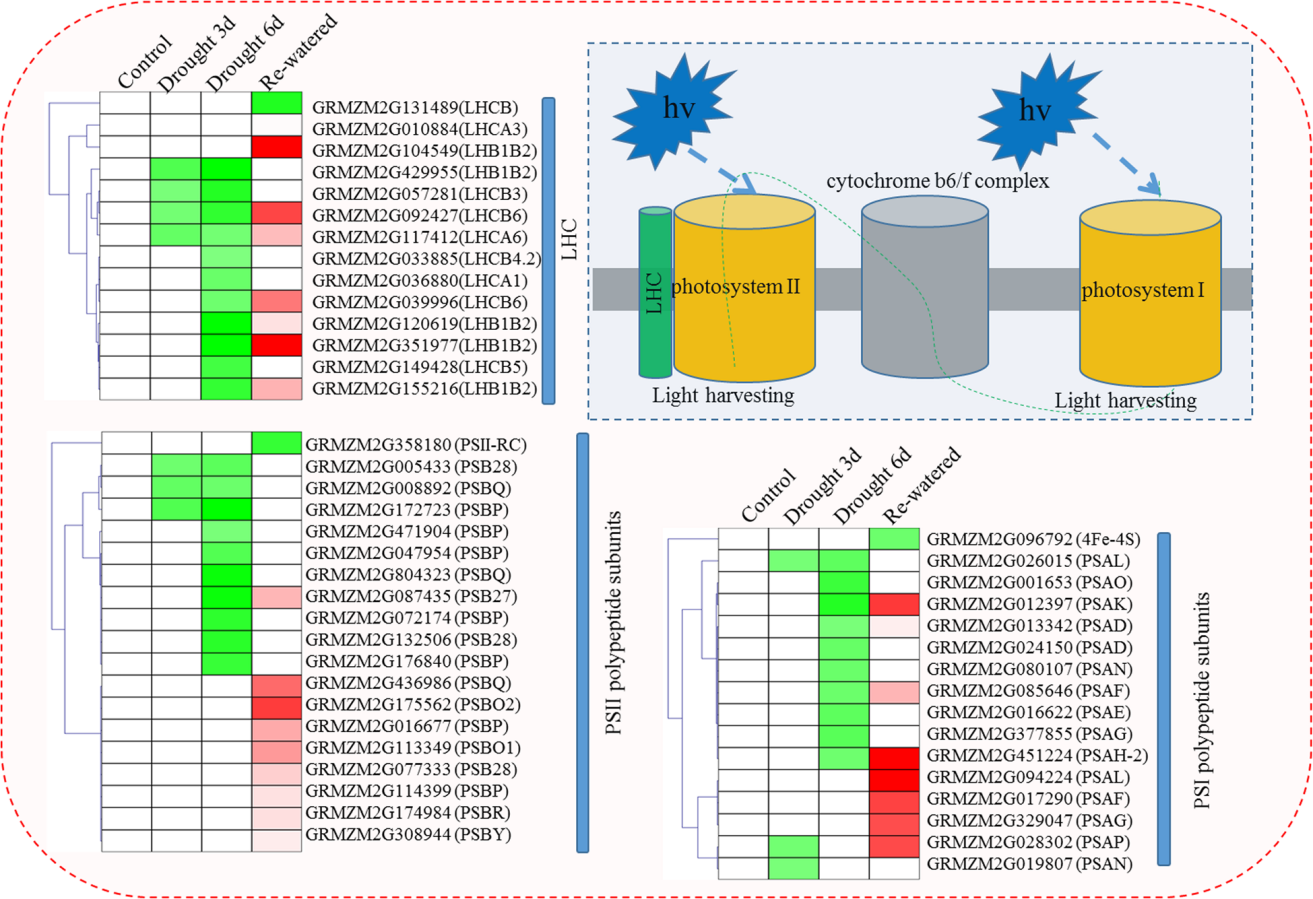
Expression patterns of the differentially expressed genes involved in various photosynthetic systems. Unigenes were enriched in different photosynthetic pathways related to the light-harvesting complex (LHC), photosystem II (PSII), and photosystem I (PSI) at the three analyzed time-points. Red and green boxes represent up- and down-regulated differentially expressed genes, respectively. The different subunits of the LHC, PSII, and PSI are annotated. The gene expression levels before initiating the drought treatment were set as controls. The map was generated based on the log_2_ fold change values (treated/control)

**Fig. 6:**
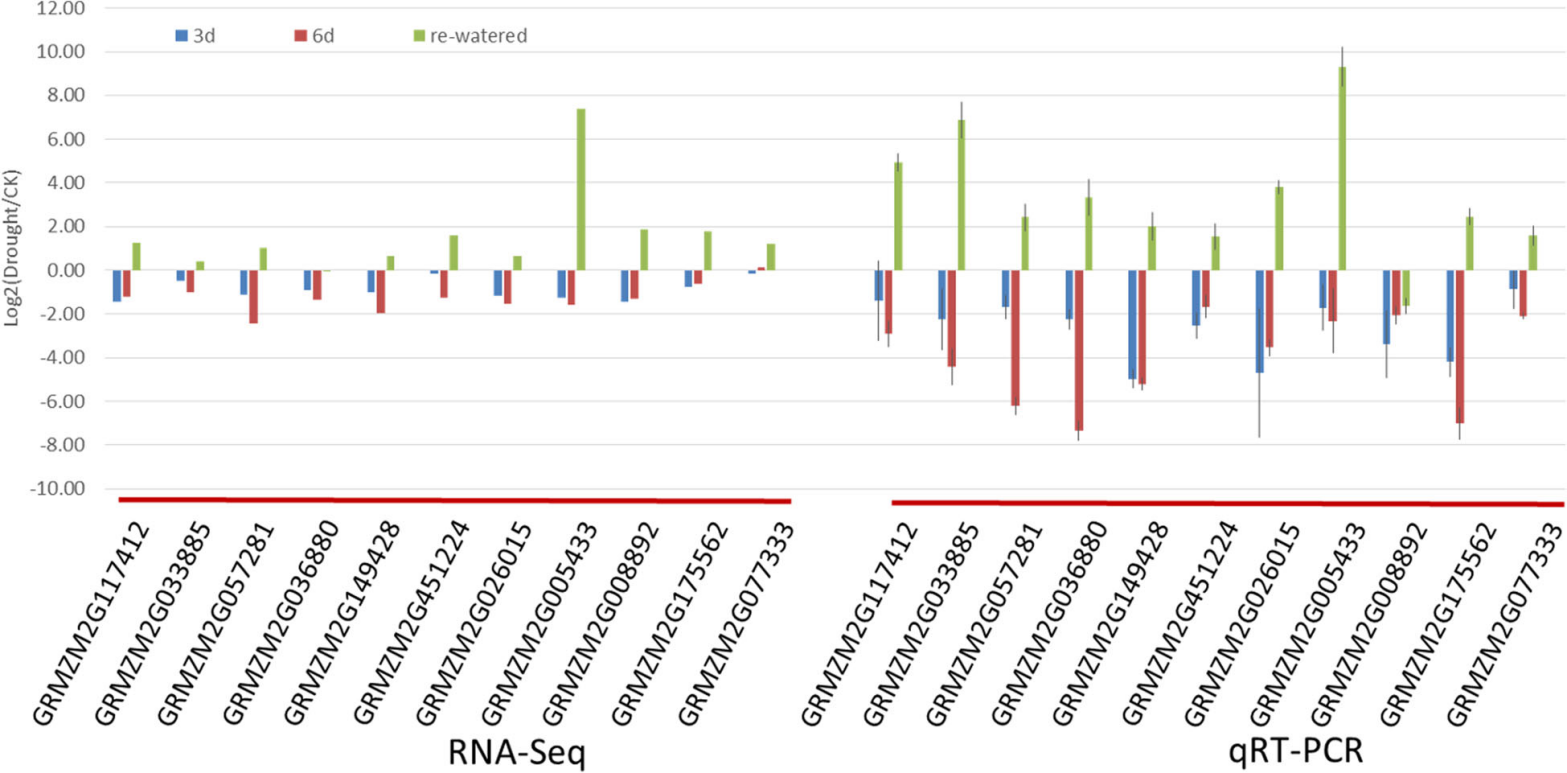
Validation of the photosynthesis-related gene expression levels. A qRT-PCR experiment was used to validate the expression patterns of genes related to photosynthetic systems

Photosynthesis is affected by drought stress [13]. We also observed a lower photosynthetic rate and lower efficiency of PSII electron transport in the drought seedlings (Fig. 1f-i). Next, we analyzed the DEGs related to photosynthesis (Fig. 5) and determined that the expression levels of two PSI genes, namely a *PSAL*-encoding gene (GRMZM2G026015) and a PSAN-encoding gene (GRMZM2G019807), were significantly down-regulated after the 3-day drought treatment. Additionally, the expression levels of 10 genes, including those encoding subunits from photosystem I and II as *PSAL, PSAO, PSAG, PSAD, PSAN, and PSAE*, were down-regulated after the 6-day drought treatment. Interestingly, in re-watered plants, the expression levels of most of these genes returned to control levels, while the expression of some genes was up-regulated. For example, the expression levels of GRMZM2G012397 (*PSAK*) and GRMZM2G451224 (*PSAH-2*) were considerably decreased at the 6-day drought treatment, but were induced during the water recovery period. A similar expression trend was observed for PSII genes. Specifically, the expression levels of many genes, including those encoding *PSB28* (GRMZM2G005433), *PSBQ* (GRMZM2G008892), and *PSBP* (GRMZM2G172723), were down-regulated by drought conditions, particularly after the 6-day treatment. The expression of most of these genes recovered to control levels in re-watered plants. For the LHC, the expression levels of four genes (i.e., GRMZM2G429955, GRMZM2G057281, GRMZM2G092427, and GRMZM2G117412) were down-regulated after 3 days of drought conditions. The expression levels of more than 10 LHC genes were down-regulated after the 6-day drought treatment. Following the water recovery period, the expression levels of most of these LHC genes returned to control levels or were up-regulated. To further confirm the accuracy of these results, the photosynthetic pathway gene expression levels were validated using qRT-PCR (Fig. 6).

**Fig. 7:**
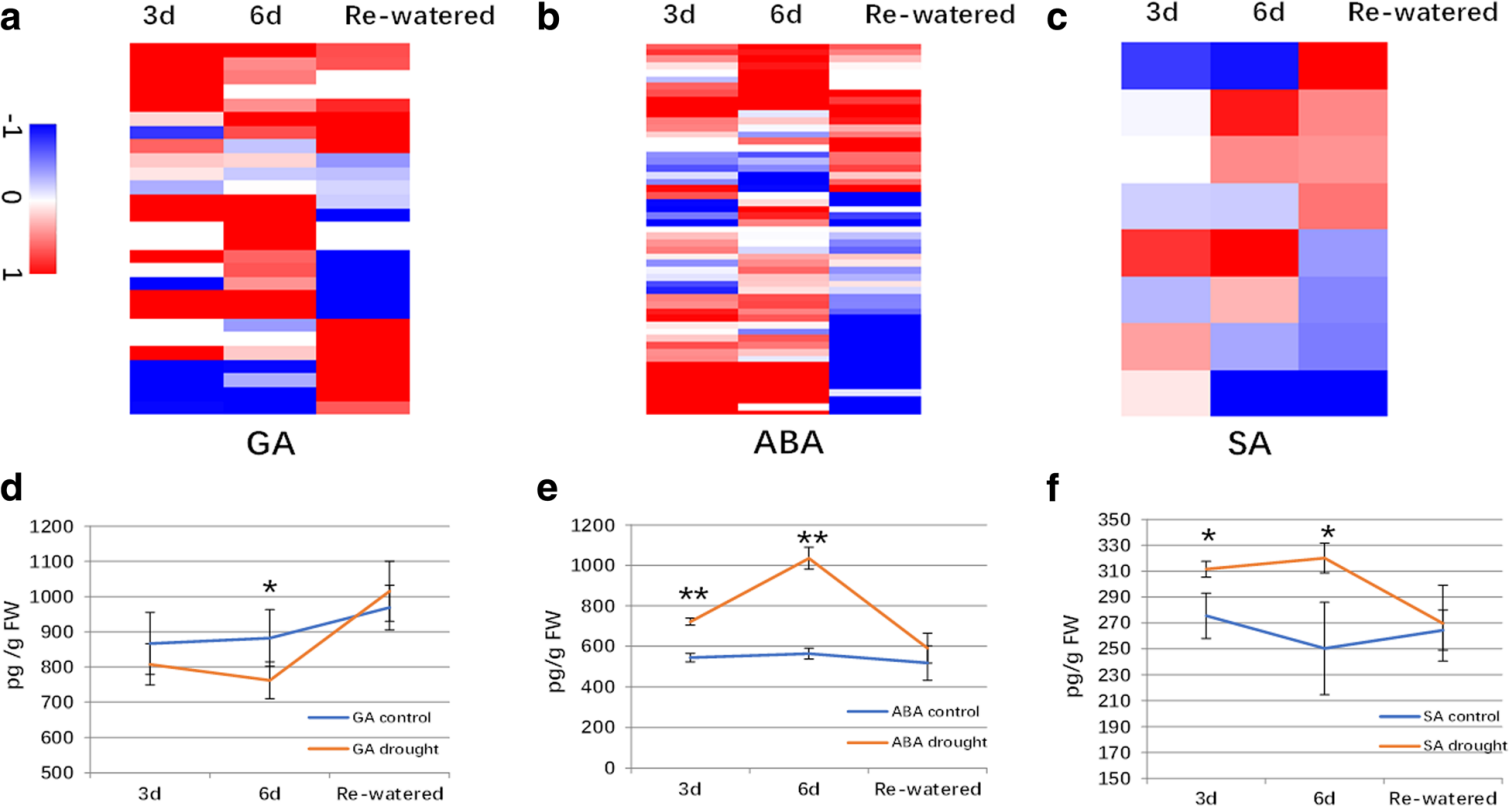
Expression patterns of genes involved in hormone metabolism and the amounts of hormones in seedlings. Expression patterns of genes involved in the metabolism and synthesis of Gibberellin (**a**), Abscisic Acid (**b**), and Salicylic Acid (**c**) were analyzed. The heat map was generated using log_2_(fold change; treated/control) values, and the up- and down-regulated genes are indicated in red and blue, respectively. The amount of Gibberellin (**d**), Abscisic Acid (**e**), and Salicylic Acid (**f**) were measured in both control (blue line) and drought treatment seedlings (orange line). Significant values were indicated as asterisks (*: <=0.05; ** < =0.01)

Hormones are important drought-responsive factors [38, 39]. Thus, we investigated the expression patterns of hormone-related genes under drought and water recovery conditions. A total of 92 hormone-related genes involving in metabolisms of gibberellin (28/54), salicylic acid (8/24) and abscisic acid (56/103) were identified at the three time-points (Fig. 7a-c). A majority of these genes (50/84, fold change> = 1.5) were up-regulated by under drought treatment, especially after 6 days drought treatment. In addition, we also measured the amount of the three hormones in the three stages (Fig. 7d-f). GA content was decreased at 3d and 6d, especially at 6d (*p* = 0.048), but recovered to control level at re-watered stage. Content of ABA was strongly induced by drought treatments at 3d (*p* = 1.7*10^− 4^) and 6d (*p* = 8.9*10^− 5^), especially at 6d, and decreased after re-watering. SA showed no significant difference in response to the drought treatments as well as water recovery. These results suggested that GA and ABA production might be involved in drought treatment and water recovery.

### Changes to the expression levels of genes related to cell wall development

The leaves of drought treatment seedlings were shorter than control seedlings. Genes involved in cell wall development might be affected during drought treatment stage. So we focused on three types of genes, which were annotated as Cellulose, expansion and xyloglucan endotransglucosylase, respectively. Most of these genes were down-regulated during drought treatment stages (see Additional file 2: Figure S8), especially at 6d. Interestingly, genes annotated with expansion and xyloglucan endotransglucosylase were up-regulated at re-watered stage. This might explain the relatively normal phenotype of the re-watered seedlings. These results indicated that genes involved in cell wall development were down-regulated during drought treatment stages, which result in the relative shorter leaves.

## Discussion

In the present study, we conducted a comparative physiological and transcriptomic analysis under drought stress and water recovery conditions. Leaf growth was repressed by water deficit stress. To survive the drought conditions, plants required multiple plant metabolic pathways that decreased gas exchange and photosynthetic efficiency and ultimately prevented plants from senescing. Additionally, we identified many DEGs responsive to drought stress through RNA-seq analysis, and summarized the associated GO terms. Our results imply that seedlings rely on complex biological processes to counter drought stress.

### Multiple physiological responses under water deficit stress: Water content, leaf rolling, leaf expansion and photosynthesis-related traits

The closing of stomata is a well-known mechanism plants use to avoid water loss in response to drought stress, but this adaptation also results in decreased CO_2_ assimilation and lower photosynthetic efficiency [40]. Under water deficit conditions, cell division and dry matter accumulation reportedly decrease because of inhibited light harvesting [20, 22]. We observed greater leaf rolling with decreasing water content in drought-treated seedling leaves (Fig. 1a and b). The re-watering of plants resulted in the full expansion of leaves (Fig. 1c). However, the length and width of drought-treated leaves were significantly shorter than that of control leaves, which might be the result of significant decreases in photosynthesis-related parameters, including photosynthetic rate, chlorophyll content and fluorescence. Our findings are consistent with those of a previous study that concluded photosynthesis, carbohydrate metabolism, and cell division [36] are inhibited under drought conditions [20]. Although leaf wilting was eliminated in drought-treated plants by re-watering, the mean values of photosynthesis-related parameters, such as Fv/Fm and ΦPSII, during the water recovery period remained lower than those of the control (Fig. 1f-i) plants. It is possible that the damages to the photosystem induced by drought stress were too severe, or the recovery time was insufficient. These observations imply that the physiological water content may be tightly correlated with the water content of seedling leaves. This may be useful for evaluating photosynthetic activities under drought conditions.

### Transcription factors are important for drought stress responses

Because TFs can coordinately regulate the expression of many downstream genes, they are considered to have important roles during responses to various abiotic stresses. The genome-wide gene expression patterns induced by drought stress have been characterized for various TFs in maize, including the NAC [1], WRKY and VQ [11], bZIP [41], and CCH-type zinc finger [42] families. Most of these TFs are transcriptionally responsive to water deficits, as indicated by our results (Fig. 2c and d). The NAC family, which contains 37 members, was one of the largest TF families whose genes were differentially expressed in response to drought conditions (see Additional file 2: Figure S5). *ZmNAC111* (GRMZM2G127379) was reported to be highly responsive to drought stress at seedling stage [1], and the over-expression of *ZmNAC111* resulted in increased drought tolerance. Our RNA-seq data revealed that *ZmNAC111* expression was sharply up-regulated by the 3-day and 6-day drought treatments (see Additional file 7). The FPKM values after the 3-day and 6-day drought treatments were 38.80 and 100.03, respectively. These values then decreased and remained low after plants re-watered (i.e., FPKM value of 5.43). *NLP7* encodes a RWP-RK transcription factor, and decreased expression levels of this gene may contribute to enhanced drought tolerance in *A. thaliana* [43]. According to our RNA-seq data, the expression of the *AtNLP7* paralog in maize (i.e., GRMZM2G048582; named as ZmNLP7) was down-regulated during the drought and water recovery periods (see Additional file 8).

TFs regulate plant physiological change by targeting downstream genes to achieve stress response. *ZmGOLS2*, which encodes a galactinol synthase, confers drought tolerance [27]. Its expression is regulated by *ZmDREB2A*, which belongs to the DREB TF family. The up-regulated expression of *ZmVPP1*, which encodes a vacuolar H^+^ pyrophosphatase, was recently observed to increase drought tolerance. The three detected MYB TF family *cis*-elements may be contributing factors for the enhanced drought tolerance [23]. We previously reported that the expression levels of WRKY and VQ TF genes were up-regulated in response to drought stress. The up-regulated expression levels were cross-linked, and many drought-responsive elements were detected in the corresponding promoter regions based on our RNA-seq data [11]. These results imply that over-expressing or knocking down DEGs through genetic manipulations may increase drought tolerance in maize plants.

### Photosynthesis-related processes are sensitive to water deficit and recovery

Of the many biological processes activated when plants encounter environmental stresses [12, 44, 45], the photosynthesis-related processes are the most sensitive to water deficit and recovery [13, 20, 22]. Thus, photosynthetic parameters have been universally used to evaluate plant drought tolerance [46, 47]. A detailed analysis of our RNA-seq data indicated that 15, 19, and 14 genes related to PSI, PSII, and the LHC, respectively, were differentially expressed depending on the treatments. The expression levels of most of these genes were down-regulated under water deficit conditions, and up-regulated during the water recovery period. These results were consistent with the phenotypic changes regarding photosynthetic efficiency, chlorophyll fluorescence, and SPAD values (Fig. 1). Photosynthetic activity is considered the main yield-determining factor, especially in plants exposed to abiotic stresses [48]. Ribulose-1, 5-bisphosphate carboxylase/oxygenase (Rubisco) activities are associated with photosynthetic rate-limiting steps, especially under environmental stress conditions [48–50]. A comprehensive analysis of our RNA-seq data revealed that the expression levels of seven Rubisco-related genes were sharply decreased in response to drought stress (see Additional file 5). In particular, the gene encoding the Rubisco small subunit (i.e., GRMZM2G113033) was differentially expressed at all three analyzed time-points. Additional analyses of metabolic pathways indicated that the light reaction was more sensitive to water stress than the other factors (see Additional file 2: Figure S7). Thus, photosynthetic changes occurred soon after plants were exposed to drought conditions. Additionally, photosynthetic efficiency decreased, which resulted in the development of smaller seedlings relative to the well-watered control plants.

We also observed that most of the photosynthesis-related genes were more highly expressed in the drought-stressed seedlings than in the controls following the water recovery period. Interestingly, the values for the corresponding photosynthesis-related parameters (i.e., photosynthetic efficiency, SPAD values, and chlorophyll fluorescence) in the drought-stressed seedlings did not exceed the control values. It is possible that compensatory mechanisms were activated after the water recovery period in plants exposed to long-term drought conditions. The time required for re-watered seedlings to fully recover may be dependent on the severity of the drought stress exposure. Additional studies are required to fully characterize these physiological responses and the underlying mechanisms.

### Multiple biological processes are involved in drought stress responses according to GO analysis

Many studies have concluded that reactive oxygen species (ROS) accumulate in dehydrated plants [20, 51–53]. Multiple biological processes (e.g., oxidation/reduction, oxidoreductase activities, transferase activities, and carbohydrate metabolic processes) were activated by changing gene expression patterns coordinately in respond to ROS accumulation [13, 20]. Additionally, low ROS doses have positive effects on cell protection, while high doses lead to programmed cell death in plants [54]. Our results indicate that the three main biological processes activated by the 3-day drought treatment were related to stimulus responses, oxidoreductase activities, and the cell membrane. After the 6-day drought treatment, many DEGs were related to the oxygen-evolving complex, stimulus responses, and oxidoreductase activities. One of the significant cellular carbohydrate metabolic process (GO:0044262) was enriched at all three investigated time-points. However, many biological processes, DEGs, and the corresponding phenotypes that were affected by the drought treatments recovered when watering was resumed. These processes increased electron leakage to triplet oxygen, resulting in programmed cell death [55], which indirectly affected cell proliferation under drought conditions. Our phenotyping analysis revealed that although most drought-treated seedlings fully recovered after the water recovery period, some cells at the leaf tips had died (Fig. 1) and the exposure to drought stress decreased overall plant size.

### Carbohydrate metabolism is important for the survival of drought-treated seedlings

Carbohydrate metabolism is one of the most important plant processes for absorbing the energy generated during photosynthesis, and its substrates have been reported to be involved in drought stress responses in addition to acting as energy sources. Changes to the expression of genes associated with carbohydrate metabolism alter the carbohydrate contents of different tissues. Additionally, drought stress also induces the accumulation of different sugars, including glucose [13, 20]. The ectopic expression of genes related to carbohydrate metabolism improves drought tolerance in maize [4] and rice [29]. Our data revealed that the expression of GRMZM2G139300, which encodes a cell wall enzyme that hydrolyzes sucrose into glucose and fructose, was up-regulated after the 6-day drought treatment (i.e., FPKM: drought/control was 83.50/18.36), suggesting that the sucrose biosynthetic and metabolic pathways were induced by drought stress. According to GO analyses, most carbohydrate-related processes were enriched under drought conditions (Fig. 3), including five categories related to carbohydrate metabolism. We also observed that the expression levels of genes associated with oligosaccharide metabolism or disaccharide biosynthesis and metabolism were mainly up-regulated after the 6-day drought treatment (Fig. 3).

It has been reported that over-expression of *NLP7*, encoding RWP-RK transcription factor, in transgenic tobacco plants resulted in enhanced carbon and nitrogen assimilation as well as an elevated photosynthetic rate [56, 57], implying that the activities of the carbohydrate and nitrogen metabolic pathways are coordinated. In our data, we also observed that several processes related to carbon and nitrogen metabolism and biosynthesis were over-represented during GO analyses. In addition, some nitrogen metabolism-related candidate genes belonging to the RWP-RK TF family were differentially expressed. We concluded that carbohydrate and nitrogen metabolic activities were repressed under drought conditions, which resulted in carbon and nitrogen deficiencies. The insufficient carbon and nitrogen levels considerably affected chloroplast development, which led to lower SPAD values. In other words, modulating the expression of genes influencing carbohydrate or nitrogen metabolic pathways may be a viable option for enhancing drought tolerance in maize seedlings.

## Conclusions

We herein describe the results of our comprehensive investigation regarding physiological responses and gene expression patterns in plants treated with drought stress and a water recovery period. Phenotypic measurements suggested that water deficit stress decreased the photosynthetic efficiency and inhibited cell division, resulting in the production of relatively small seedling leaves. More than 6000 DEGs were detected through RNA-seq analysis, with many different TF families identified as sensitive to drought stress. Among the DEGs, the expression levels of more than 30 genes related to photosynthetic systems were down-regulated under drought conditions, which was consistent with the corresponding phenotypic variations in chlorophyll fluorescence, SPAD values, and photosynthetic efficiency. The results of GO analyses revealed that many drought-responsive pathways, including those related to carbohydrate and nitrogen metabolism, were induced by drought conditions. The amount of GA was decreased during drought treatments, specifically at 6d and showed no significance difference at the re-watered stage. However, ABA showed opposite pattern in comparison with that of GA. So, GA and ABA might participate in drought response to regulate plant growth. Most of genes related to cell wall development also exhibited down-regulation, which best explain the phenotype of relative small leaves. Taken together, our findings might serve as a useful resource for future investigations of the specific functions of these drought-responsive genes.

## Additional files

Additional file 1: Premiers used in the validation of RNA-seq data by means of qRT-PCR. (PDF 99 kb)

Additional file 2: **Figure S1.** Elongation rate of leaf during drought treatment stage(3d-6d) and Re-watered stage. The elongation rate of leaf during drought stage (3d-6d) and re-watered stage for control and drought treatment seedlings were calculated respectively. The X-axis represents two different stages (drought stage from 3d to 6d and re-watered stage from 6d to re-watered). The Y-axis represents the elongation rate of leaf (elongation length of leaf divided by elongation days). Orange line represents drought treatment seedlings and blue line represents control seedlings. **Figure S2.** Global gene expression level. Number of expressed genes, including those encoding transcription factors (TFs), and their expression levels (median boxplot) after a 3-day or 6-day drought treatment and a 1-day water recovery period according to RNA sequencing data (**A** and **B)**. **Figure S3.** PCA analysis of six samples in the three time points. We cluster the six seedling samples in the three time points using R function princomp with expression value(FPKM) as input. Green points show control seedlings and black color show drought treatment seedlings. **Figure S4.** Correlation analysis between two biological replicates under drought treatments and controls. We calculated the expression level (FPKM) for each replicate. The normalized data of log2 (FPKM + 1) was used to calculate the Pearson Correlation Coefficient (PCC). **Figure S5.** Number of differentially expressed transcription factor genes in response to drought stress treatments. The different transcription factor families responding to water deficit and recovery were summarized and compared. The X-axis represents the transcription factor family members. The Y-axis represents the number of differentially expressed family members. **Figure S6.** The identification of Co-expressing genes with *ZmNAC111* Using qTeller data and our drought-treatment data. We selected the top 100 co-expressing genes with *ZmNAC111* (measure by Pearson correlation coefficient) using public qTeller data and our data, respectively. The overlapped genes were considered as the target genes of *ZmNAC111*. **Figure S7.** Overview of different metabolic pathways and carbohydrates affected by the differentially expressed genes in response to drought and water recovery treatments. Metabolic pathways associated with differentially expressed genes after the 3-day drought treatment (**A**), 6-day drought treatment (**B**), and water recovery period (**C**). Carbohydrate metabolic pathways associated with differentially expressed genes after the 3-day drought treatment (**D**), 6-day drought treatment (**E**), and water recovery period (**F**). In each panel, the expression levels of up- and down-regulated genes are indicated in blue and red, respectively. **Figure S8.** Expression profile of genes involved in cell wall development. Cellulose, expansin and xyloglucan endotransglucosylase related genes are involved in cell wall development. The fold change value (log_2_(MT/WT)) in the figure is calculated. Green color represents down-regulated genes. Red color represents up-regulated genes. (PDF 1382 kb)

Additional file 3: Summary of reads, mapped to B73 reference genome, yielded by illumina sequencing technique. (XLSX 9 kb)

Additional file 4: Up regulated genes at 3 day, 6 day and re-watered stages. (XLSX 194 kb)

Additional file 5: Down regulated genes at 3 day, 6 day and re-watered stages. (XLSX 268 kb)

Additional file 6: The overlap DEGs between maize seedlings and silk, ovary tissues. (XLSX 20 kb)

Additional file 7: Up regulated TFs at 3 day, 6 day and re-watered stages. (XLSX 24 kb)

Additional file 8: Down regulated TFs at 3 day, 6 day and re-watered stages. (XLSX 23 kb)

Additional file 9: Enriched GO terms among the DEGs in response to water deficit and recovery. (XLSX 20 kb)

Additional file 10: Numbers of DEGs in different metabolisms in response to water deficit and recovery. (XLSX 9 kb)

## List of abbreviations

DEG: Differentially expressed gene
PCR: Polymerase chain reaction
TF: Transcriptional factor

## References

[1] Mao H, Wang H, Liu S, Li Z, Yang X, Yan J, Li J, Tran LS, Qin FA (2015). Transposable element in a NAC gene is associated with drought tolerance in maize seedlings. Nat Commun.

[2] Nelson DE, Repetti PP, Adams TR, Creelman RA, Wu J, Warner DC, Anstrom DC, Bensen RJ, Castiglioni PP, Donnarummo MG (2007). Plant nuclear factor Y (NF-Y) B subunits confer drought tolerance and lead to improved corn yields on water-limited acres. Proc Natl Acad Sci U S A.

[3] Mittler R (2006). Abiotic stress, the field environment and stress combination. Trends Plant Sci.

[4] Nuccio ML, Wu J, Mowers R, Zhou H, Meghji M, Primavesi LF, Paul MJ, Chen X, Gao Y, Haque E (2015). Expression of trehalose-6-phosphate phosphatase in maize ears improves yield in well-watered and drought conditions. Nat Biotechnol.

[5] Boyer J (1982). Plant productivity and environment. Science.

[6] Kakumanu A, Ambavaram MM, Klumas C, Krishnan A, Batlang U, Myers E, Grene R, Pereira A (2012). Effects of drought on gene expression in maize reproductive and leaf meristem tissue revealed by RNA-Seq. Plant Physiol.

[7] Chen D, Wang S, Cao B, Cao D, Leng G, Li H, Yin L, Shan L, Deng X (2015). Genotypic variation in growth and physiological response to drought stress and re-watering reveals the critical role of recovery in drought adaptation in maize seedlings. Front Plant Sci.

[8] Gong F, Wu X, Zhang H, Chen Y, Wang W (2015). Making better maize plants for sustainable grain production in a changing climate. Front Plant Sci.

[9] Maiti RK, Satya P (2014). Research advances in major cereal crops for adaptation to abiotic stresses. GM Crops Food.

[10] Bänziger M, Edmeades GO, Beck D, Bellon M. Breeding for Drought and Nitrogen Stress Tolerance in Maize- From Theory to Practice. International Maize and Wheat Improvement Center; 2000. pp. 6-8.

[11] Song W, Zhao H, Zhang X, Lei L, Lai J (2016). Genome-wide identification of VQ motif-containing proteins and their expression profiles under abiotic stresses in maize. Front Plant Sci.

[12] Lei L, Shi J, Chen J, Zhang M, Sun S, Xie S, Li X, Zeng B, Peng L, Hauck A (2015). Ribosome profiling reveals dynamic translational landscape in maize seedlings under drought stress. Plant J.

[13] Min H, Chen C, Wei S, Shang X, Sun M, Xia R, Liu X, Hao D, Chen H, Xie Q (2016). Identification of drought tolerant mechanisms in maize seedlings based on transcriptome analysis of recombination inbred lines. Front Plant Sci.

[14] Campos H, Cooper M, Habben JE, Edmeades GO, Schussler JR (2004). Improving drought tolerance in maize: a view from industry. Field Crop Res.

[15] Bruce WB, Edmeades GO, Barker TC (2002). Molecular and physiological approaches to maize improvement for drought tolerance. J Exp Bot.

[16] Gall H, Philippe F, Domon J, Gillet F, Pelloux J, Rayon C (2015). Cell Wall metabolism in response to abiotic stress. Plants.

[17] Kadioglu A, Terzi R, Saruhan N, Saglam A (2012). Current advances in the investigation of leaf rolling caused by biotic and abiotic stress factors. Plant Sci.

[18] Qin F, Kakimoto M, Sakuma Y, Maruyama K, Osakabe Y, Tran LS, Shinozaki K, Yamaguchi-Shinozaki K (2007). Regulation and functional analysis of ZmDREB2A in response to drought and heat stresses in Zea mays L. Plant J.

[19] Bunce JA (2010). Leaf transpiration efficiency of some drought-resistant maize lines. Crop Sci.

[20] Hayano-Kanashiro C, Calderon-Vazquez C, Ibarra-Laclette E, Herrera-Estrella L, Simpson J (2009). Analysis of gene expression and physiological responses in three Mexican maize landraces under drought stress and recovery irrigation. PLoS One.

[21] Almoguera C, Prietodapena P, Personat JM, Tejedorcano J, Lindahl M (2012). Protection of the photosynthetic apparatus from extreme dehydration and oxidative stress in seedlings of transgenic tobacco. PLoS One.

[22] Huo Y, Wang M, Wei Y, Xia Z (2016). Overexpression of the maize psbA gene enhances drought tolerance through regulating antioxidant system, photosynthetic capability, and stress defense gene expression in tobacco. Front Plant Sci.

[23] Wang X, Wang H, Liu S, Ferjani A, Li J, Yan J, Yang X, Qin F (2016). Genetic variation in ZmVPP1 contributes to drought tolerance in maize seedlings. Nat Genet.

[24] Xiang Y, Sun X, Gao S, Qin F, Dai M (2016). Deletion of an endoplasmic reticulum stress response element in a ZmPP2C-A gene facilitates drought tolerance of maize seedlings. Mol Plant.

[25] Zhao T, Martin D, Meeley RB, Downie B (2004). Expression of the maize GALACTINOL SYNTHASE gene family: (II) kernel abscission, environmental stress and myo-inositol influences accumulation of transcript in developing seeds and callus cells. Physiol Plantarum.

[26] Gu L, Han Z, Zhang L, Downie B, Zhao T (2013). Functional analysis of the 5′regulatory region of the maize GALACTINOL SYNTHASE2 gene. Plant Sci.

[27] Gu L, Zhang Y, Zhang M, Li T, Dirk LM, Downie B, Zhao T (2016). ZmGOLS2, a target of transcription factor ZmDREB2A, offers similar protection against abiotic stress as ZmDREB2A. Plant Mol Biol.

[28] Mostofa MG, Hossain MA, Fujita M, Physiological TLP (2015). Biochemical mechanisms associated with trehalose-induced copper-stress tolerance in rice. Sci Rep.

[29] Mostofa MG, Hossain MA, Fujita M (2015). Trehalose pretreatment induces salt tolerance in rice (Oryza sativa L.) seedlings: oxidative damage and co-induction of antioxidant defense and glyoxalase systems. Protoplasma.

[30] Thao NP, Tran VLP (2016). Enhancement of plant productivity in the post-genomics era. Curr Genomics.

[31] Shan X, Li Y, Jiang Y, Jiang Z, Hao W, Yuan Y (2013). Transcriptome profile analysis of maize seedlings in response to high-salinity, drought and cold stresses by deep sequencing. Plant Mol Biol Rep.

[32] Opitz N, Paschold A, Marcon C, Malik WA, Lanz C (2014). Transcriptomic complexity in young maize primary roots in response to low water potentials. BMC Genomics.

[33] Trapnell C, Pachter L, Salzberg SL (2009). TopHat: discovering splice junctions with RNA-Seq. Bioinformatics.

[34] Trapnell C, Roberts A, Goff L, Pertea G, Kim D (2012). Corrigendum: differential gene and transcript expression analysis of RNA-seq experiments with TopHat and cufflinks. Nat Protoc.

[35] Thimm O, Bläsing O, Gibon Y, Nagel A, Meyer S, Krüger P, Selbig J, Müller LA, Rhee SY, Stitt M (2004). Mapman. A user-driven tool to display genomics data sets onto diagrams of metabolic pathways and other biological processes. Plant J.

[36] Nelissen H, Sun X, Rymen B, Jikumaru Y, Kojima M, Takebayashi Y, Abbeloos R, Demuynck K, Storme V, Vuylsteke M (2017). The reduction in maize leaf growth under mild drought affects the transition between cell division and cell expansion and cannot be restored by elevated gibberellic acid levels. Plant Biotechnol J.

[37] Oury V, Caldeira CF, Prodhomme D, Pichon J, Gibon Y, Tardieu F, Turc O. Is change in ovary carbon status a cause or a consequence of maize ovary abortion in water deficit during flowering? Plant Physiol. 2016;171(2):997-1008. 10.1104/pp.15.01130 PMC4902574 27208256

[38] Yamaguchi-Shinozaki K, Shinozaki K (2006). Transcriptional regulatory networks in cellular responses and tolerance to dehydration and cold stresses. Annu Rev Plant Biol.

[39] Argueso CT, Ferreira FJ, Kieber JJ (2009). Environmental perception avenues: the interaction of cytokinin and environmental response pathways. Plant Cell Environ.

[40] Assmann SM, Jegla T (2016). Guard cell sensory systems. Recent insights on stomatal responses to light, abscisic acid, and CO2. Curr Opin Plant Biol.

[41] Ying S, Zhang D, Fu J, Shi Y, Song Y, Wang T, Li Y (2012). Cloning and characterization of a maize bZIP transcription factor, ZmbZIP72, confers drought and salt tolerance in transgenic Arabidopsis. Planta.

[42] Bogamuwa SP, Jang JC (2014). Tandem CCCH zinc finger proteins in plant growth, development and stress response. Plant Cell Physiol.

[43] Castaings L, Camargo A, Pocholle D, Gaudon V, Texier Y, Boutet-Mercey S, Taconnat L, Renou J, Daniel-Vedele F, Fernandez E (2009). The nodule inception-like protein 7 modulates nitrate sensing and metabolism in Arabidopsis. Plant J.

[44] Bray EA (1997). Plant responses to water deficit. Trends Plant Sci.

[45] Seki M, Umezawa T, Urano K, Shinozaki K (2007). Regulatory metabolic networks in drought stress responses. Curr Opin Plant Biol.

[46] Osakabe Y, Osakabe K, Shinozaki K, Tran LP (2014). Response of plants to water stress. Front Plant Sci.

[47] Chaves MM, Flexas J, Pinheiro C (2008). Photosynthesis under drought and salt stress: regulation mechanisms from whole plant to cell. Ann Bot London.

[48] Niinemets U, Berry JA, von Caemmerer S, Ort DR, Parry MA, Poorter H (2017). Photosynthesis: ancient, essential, complex, diverse ... And in need of improvement in a changing world. New Phytol.

[49] Hermida-Carrera C, Kapralov MV, Galmés J. Rubisco catalytic properties and temperature response in crops. Plant Physiol. 2016; 171(4):2549-61. 10.1104/pp.16.01846 PMC4972260 27329223

[50] Galmes J, Kapralov MV, Andralojc PJ, Conesa MA, Keys AJ, Parry MA, Flexas J (2014). Expanding knowledge of the rubisco kinetics variability in plant species: environmental and evolutionary trends. Plant Cell Environ.

[51] Selote DS, Bharti S, Khanna-Chopra R (2004). Drought acclimation reduces O2-accumulation and lipid peroxidation in wheat seedlings. Biochem Bioph Res Co.

[52] Ahmad N, Malagoli M, Wirtz M, Hell R (2016). Drought stress in maize causes differential acclimation responses of glutathione and sulfur metabolism in leaves and roots. BMC Plant Biol.

[53] Asada K (1999). The water-water cycle in chloroplasts: scavenging of active oxygens and dissipation of excess photons. Annu Rev Plant Physiol Plant Mol Biol.

[54] Gechev TS, Hille J (2005). Hydrogen peroxide as a signal controlling plant programmed cell death. J Cell Biol.

[55] Gechev TS, Dinakar C, Benina M, Toneva V, Bartels D (2012). Molecular mechanisms of desiccation tolerance in resurrection plants. Cell Mol Life Sci.

[56] Coruzzi G, Bush DR (2001). Nitrogen and carbon nutrient and metabolite signaling in plants. Plant Physiol.

[57] Yu L, Wu J, Tang H, Yuan Y, Wang S, Wang Y, Zhu Q, Li S, Xiang C (2016). Overexpression of Arabidopsis NLP7 improves plant growth under both nitrogen-limiting and -sufficient conditions by enhancing nitrogen and carbon assimilation. Sci Rep.

