# Supplementary material for: Effects of drought stress and water recovery on physiological responses and gene expression in maize seedlings
Source: BMC Plant Biol. 2018 Apr 23;18:68. doi: 10.1186/s12870-018-1281-x (PMC5913800; doi:10.1186/s12870-018-1281-x)
Supplement: Supplementary file 1 — Premiers used in the validation of RNA-seq data by means of qRT-PCR. (PDF 99 kb) [file 12870_2018_1281_MOESM1_ESM.pdf]

Premiers used in the validation of RNA-seq data by means of qRT-PCR.

Gene ID	Premier name	Premier sequences (5'-3')	Product (bp)
GRMZM2G127379	F	ACGCACCAGCAAGCAATAA	176
	R	GCCGTCACTCGCTTCCTCT	
GRMZM2G171468	F	ACAAGTCGGAGGATGGCTACAG	261
	R	GACTCAAACAAAACGCCTATCA	
GRMZM2G032807	F	AGCATACCCGCAGCAAGAAC	279
	R	CCATTCCACCAACACCTCCA	
GRMZM2G048129	F	GGTGATGGTAGGGCAAAGCA	151
	R	GAATAAGTCAAACGGAGGGAGC	
GRMZM2G062531	F	CACGGCTCCTACAGGCTTCG	183
	R	GGGCTGAACGGGAAATGGT	
GRMZM2G088396	F	TTGCTGTGGCACTTTGTATCAT	214
	R	TACTGACCCTCGTCGTTATTTT	
GRMZM2G108273	F	TACAAGACGCACGAGCCG	179
	R	TCCCACAATCCCACCAG	
GRMZM2G120304	F	CCCTGGTGAACAAGGAAATG	146
	R	CGGAATGGAAACGCAAATAC	
GRMZM2G117412	F	CCAGGAGACTTTGGATTTGACC	232
	R	CCATCTGCGACACGAACAGC	
GRMZM2G033885	F	GCTGGGACTTGGTGGTTTG	110
	R	TGTATCCTCGGTCATCTCAATCT	
GRMZM2G057281	F	CCACGCAATGCTGTAGGACC	220
	R	TGCTCAGGCTTGGAGAAAGG	
GRMZM2G036880	F	GGCACAACAACATCGGAGAC	257
	R	GAGGTTAGTGGCTGAGGAAGAA	
GRMZM2G149428	F	ACCTGCTCACCGTCATCTCC	300
	R	CCAACTCAAACGCACCCTTA	
GRMZM2G451224	F	AGACGGACAAGCCCAAGCA	251
	R	AATTCGGCGGATCGTATCG	
GRMZM2G026015	F	GCTCTGGGCCTACTTTCTCC	223
	R	GAACAGGTTACATCCCAATCCA	
GRMZM2G005433	F	CCAAGCAGGGAAACAAATCA	165
	R	CGATACGCCGTGGAACAAC	
GRMZM2G008892	F	CCTCTTCAACAGCGTCACCAG	165
	R	CCGCCTTTGTATCCACGAACT	
GRMZM2G175562		GAACCAGTGCCCGACCATC	186
		TCGTCCAGCGTGTACGTGAG	
GRMZM2G077333		GCCCATCAACGAGATAGAGCC	181
		CGAGACGCACAGTCACAGGTAA	
ACTB	F	CACCTTCTACAACGAGCTCC	Yue et al,2015

	R	CAGTCAGGATCTTCATGAGG	
