# Supplementary material for: Effects of drought stress and water recovery on physiological responses and gene expression in maize seedlings
Source: BMC Plant Biol. 2018 Apr 23;18:68. doi: 10.1186/s12870-018-1281-x (PMC5913800; doi:10.1186/s12870-018-1281-x)
Supplement: Supplementary file 2 — Figure S1. Elongation rate of leaf during drought treatment stage(3d-6d) and Re-watered stage. The elongation rate of leaf during drought stage (3d-6d) and re-watered stage for control and drought treatment seedlings were calculated respectively. The X-axis represents two different stages (drought stage from 3d to 6d and re-watered stage from 6d to re-watered). The Y-axis represents the elongation rate of leaf (elongation length of leaf divided by elongation days). Orange line represents drought treatment seedlings and blue line represents control seedlings. Figure S2. Global gene expression level. Number of expressed genes, including those encoding transcription factors (TFs), and their expression levels (median boxplot) after a 3-day or 6-day drought treatment and a 1-day water recovery period according to RNA sequencing data (A and B). Figure S3. PCA analysis of six samples in the three time points. We cluster the six seedling samples in the three time points using R function princomp with expression value(FPKM) as input. Green points show control seedlings and black color show drought treatment seedlings. Figure S4. Correlation analysis between two biological replicates under drought treatments and controls. We calculated the expression level (FPKM) for each replicate. The normalized data of log2 (FPKM + 1) was used to calculate the Pearson Correlation Coefficient (PCC). Figure S5. Number of differentially expressed transcription factor genes in response to drought stress treatments. The different transcription factor families responding to water deficit and recovery were summarized and compared. The X-axis represents the transcription factor family members. The Y-axis represents the number of differentially expressed family members. Figure S6. The identification of Co-expressing genes with ZmNAC111 Using qTeller data and our drought-treatment data. We selected the top 100 co-expressing genes with ZmNAC111 (measure by Pearson correlation coefficient) using publi [file 12870_2018_1281_MOESM2_ESM.pdf]

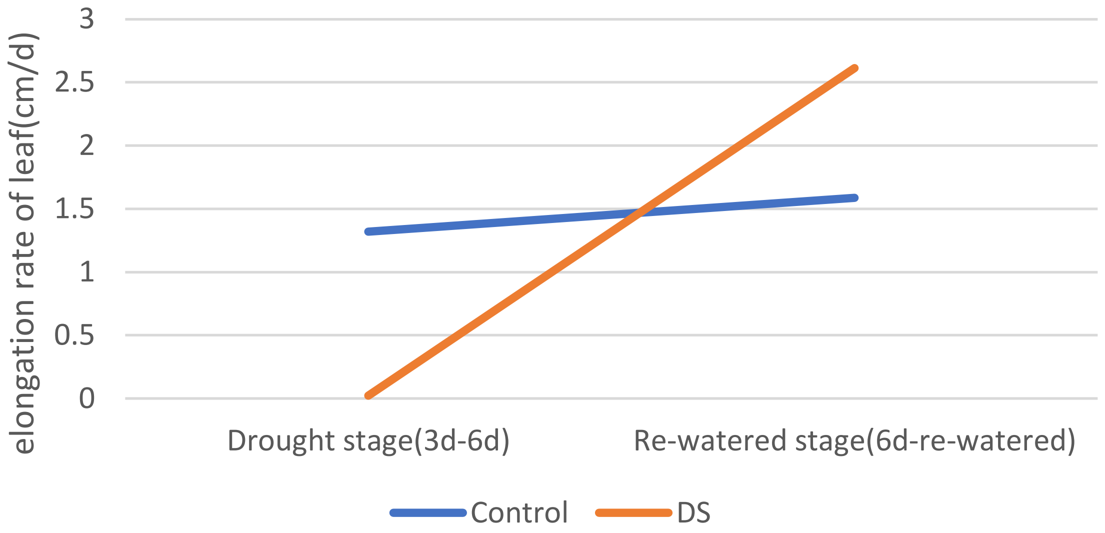

Figure S1. Elongation rate of leaf during drought treatment stage(3d-6d) and Re-watered stage. The elongation rate of leaf during drought stage (3d-6d) and re-watered stage for control and drought treatment seedlings were calculated respectively. The X-axis represents two different stages (drought stage from 3d to 6d and re-watered stage from 6d to re-watered). The Y-axis represents the elongation rate of leaf (elongation length of leaf divided by elongation days). Orange line represents drought treatment seedlings and blue line represents control seedlings.

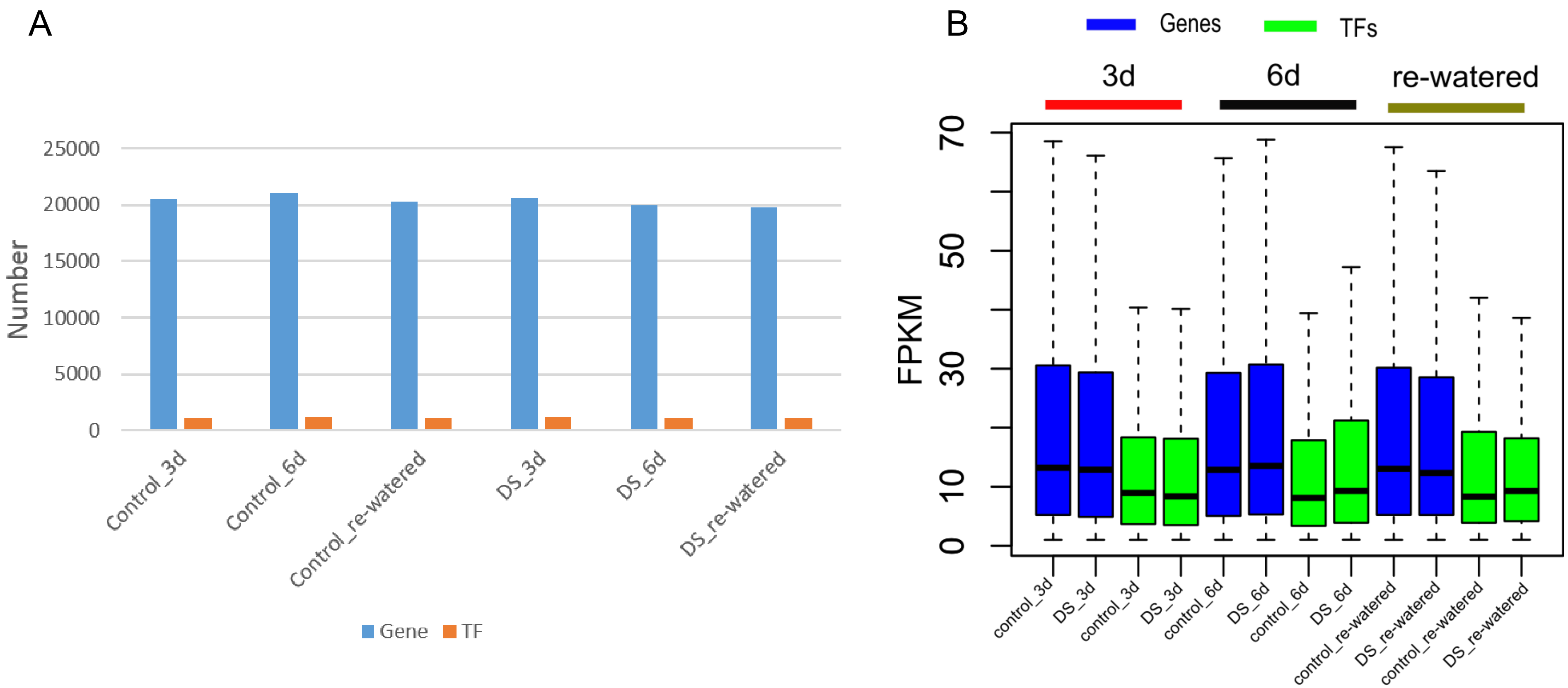

Figure S2. Global gene expression level. Number of expressed genes, including those encoding transcription factors (TFs), and their expression levels (median boxplot) after a 3-day or 6-day drought treatment and a 1-day water recovery period according to RNA sequencing data (**A** and **B**)

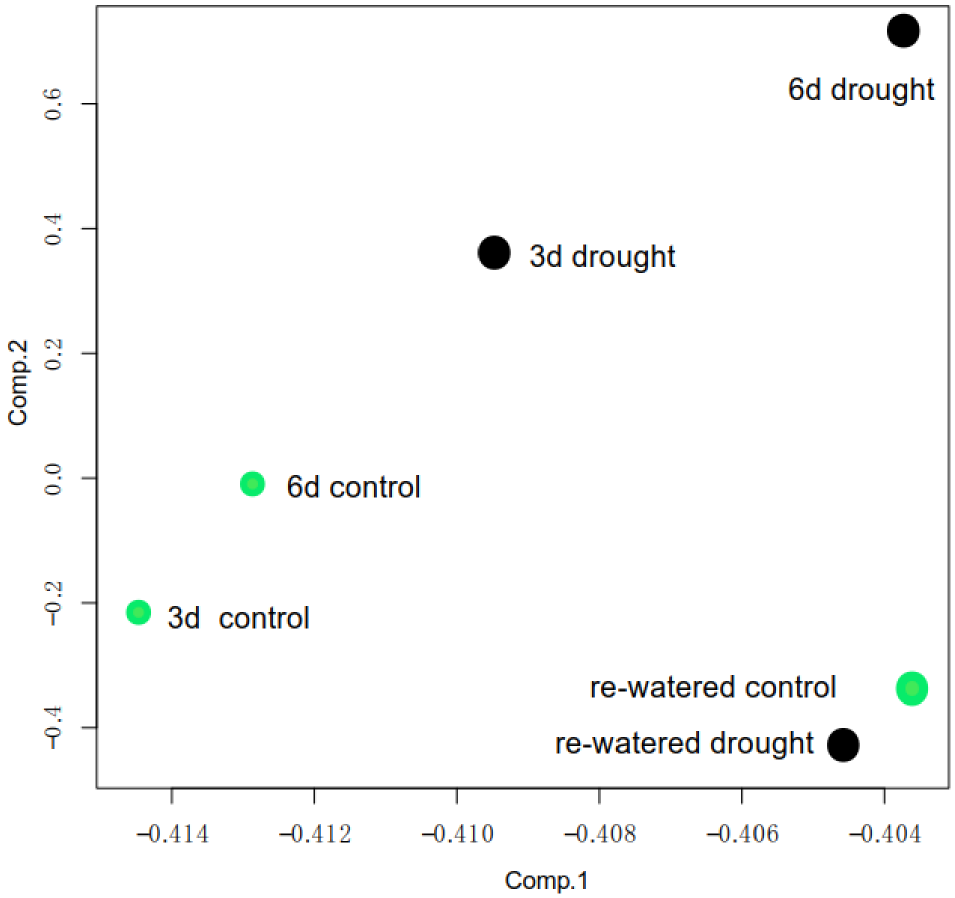

Figure S3. PCA analysis of six samples in the three time points. We cluster the six seedling samples in the three time points using R function princomp with expression value(FPKM) as input. Green points show control seedlings and black color show drought treatment seedlings.

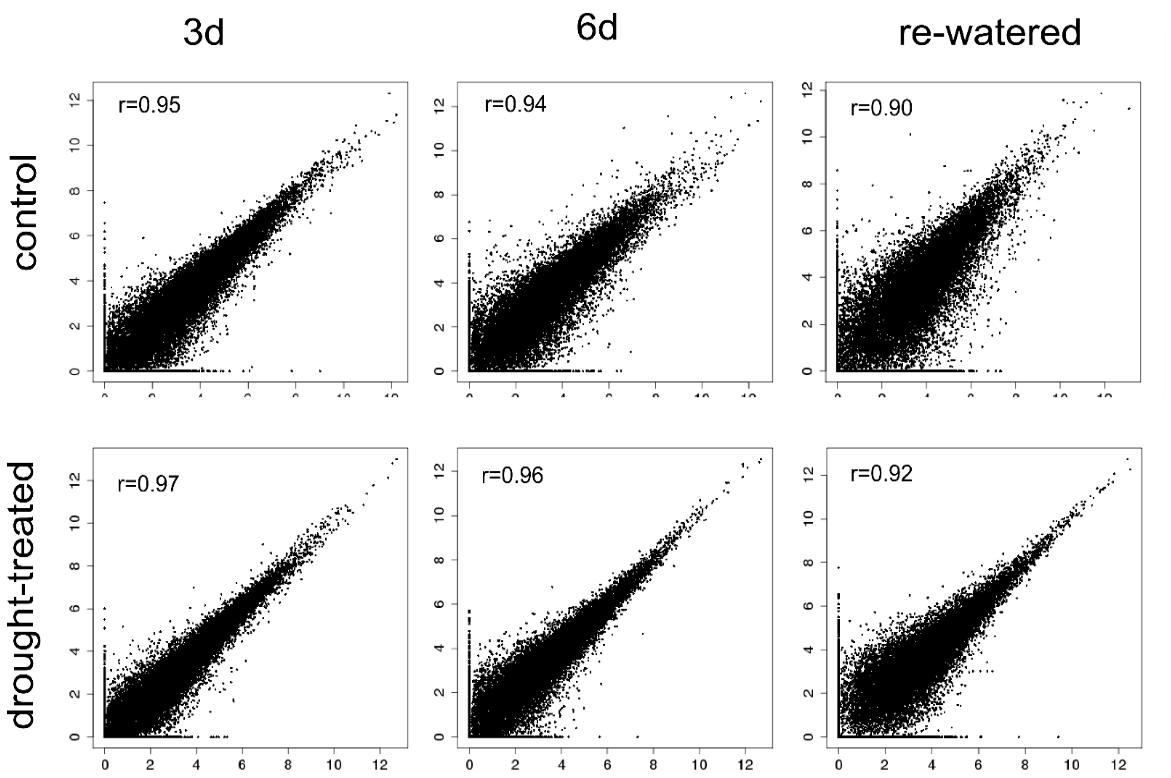

Figure S4. Correlation analysis between two biological replicates under drought treatments and controls. We calculated the expression level (FPKM) for each replicate. The normalized data of log2 (FPKM +1) was used to calculate the Pearson Correlation Coefficient (PCC).

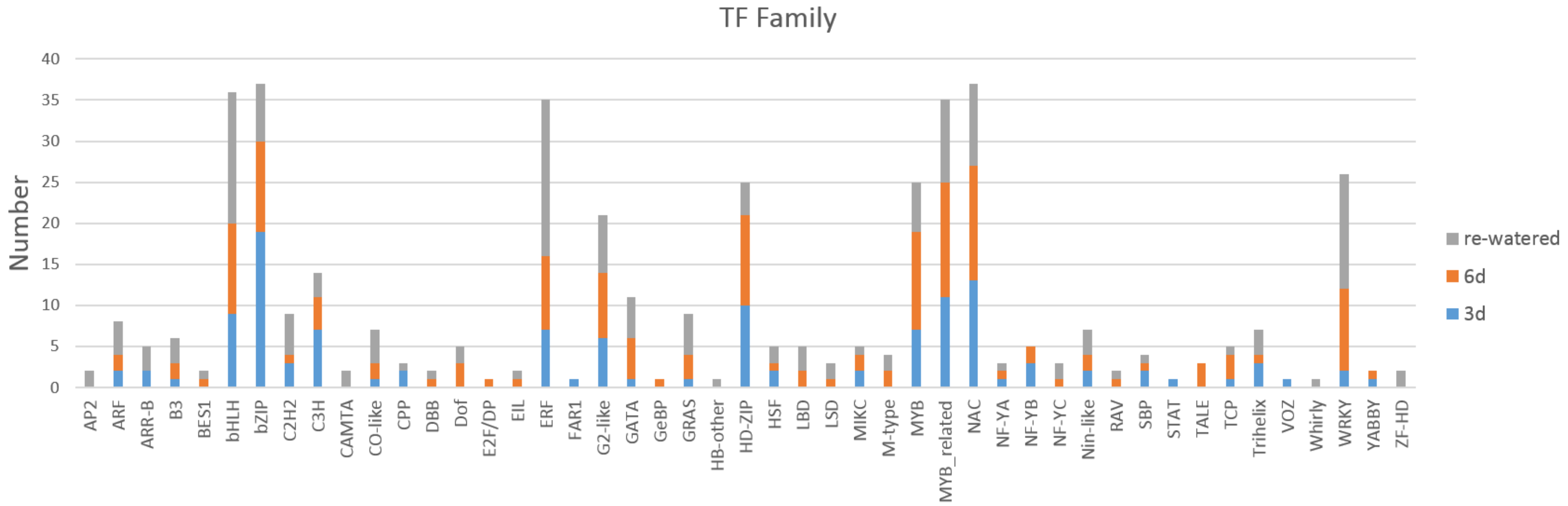

Figure S5. Number of differentially expressed transcription factor genes in response to drought stress treatments. The different transcription factor families responding to water deficit and recovery were summarized and compared. The X-axis represents the transcription factor family members. The Y-axis represents the number of differentially expressed family members.

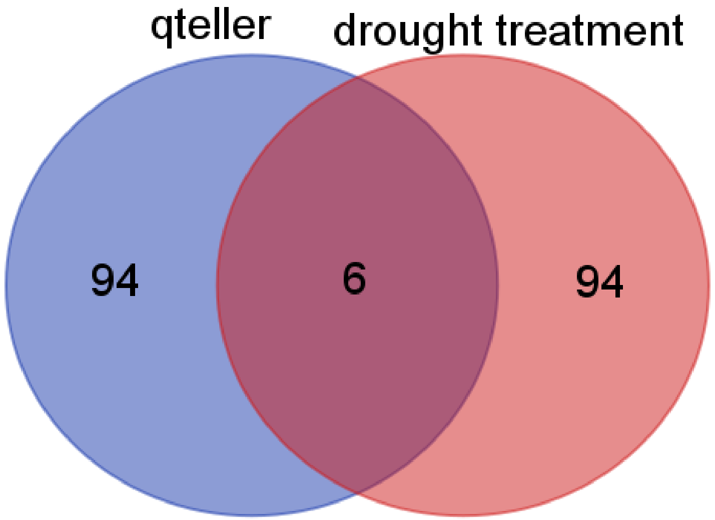

Figure S6. The identification of Co-expressing genes with *ZmNAC111* Using qTeller data and our drought-treatment data. We selected the top 100 co-expressing genes with *ZmNAC111* (measure by Pearson correlation coefficient) using public qTeller data and our data, respectively. The overlapped genes were considered as the target genes of *ZmNAC111*.

(A)

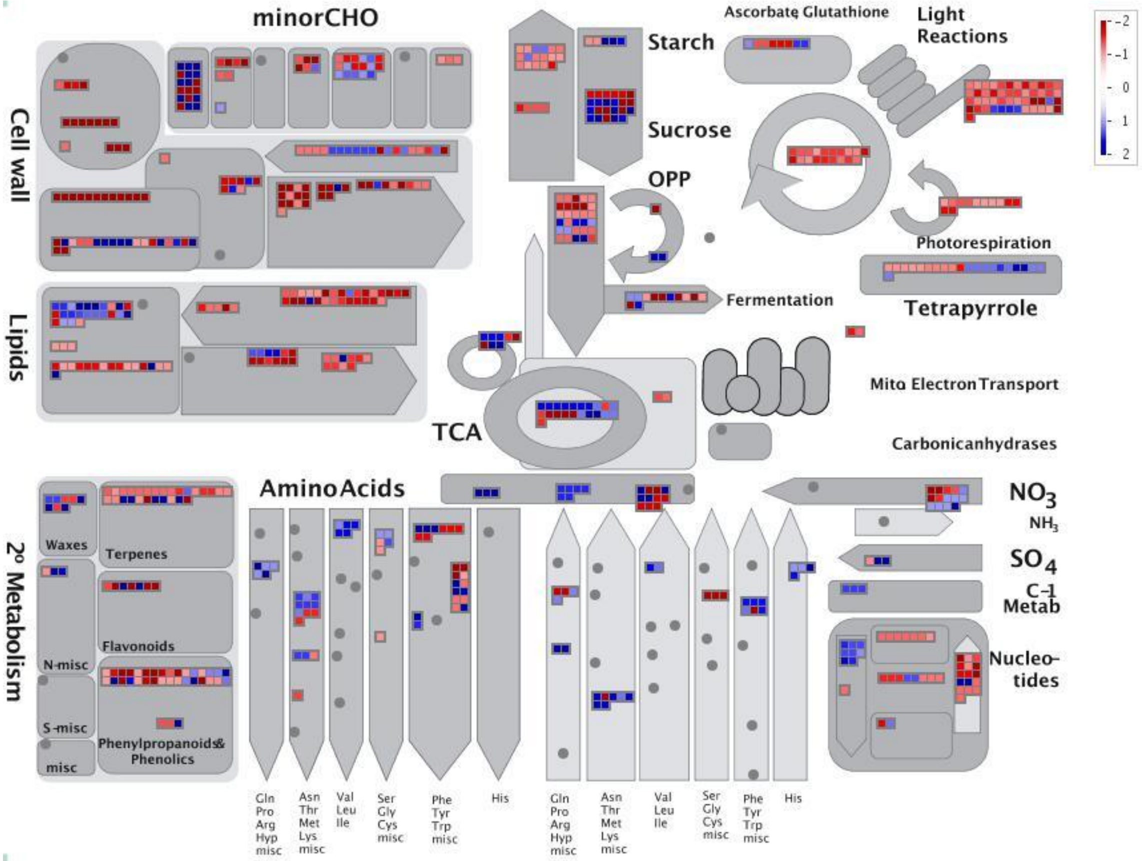
minorCHO
Cell wall
Lipids
2° Metabolism
Waxes
Terpenes
Flavonoids
N-misc
S-misc
misc
Phenylpropanoids&
Phenolics
Starch
Sucrose
OPP
Fermentation
TCA
Amino Acids
Ascorbate Glutathione
Light
Reactions
Photorespiration
Tetrapyrrole
Mito Electron Transport
Carbonicanhydrases
NO3
NH3
SO4
C-1
Metab
Nucleo-
tides
Gln
Pro
Arg
Hyp
misc
Asn
Thr
Met
Lys
misc
Val
Leu
Ile
Ser
Gly
Cys
misc
Phe
Tyr
Trp
misc
His
-2
-1
0
1
2

(B)

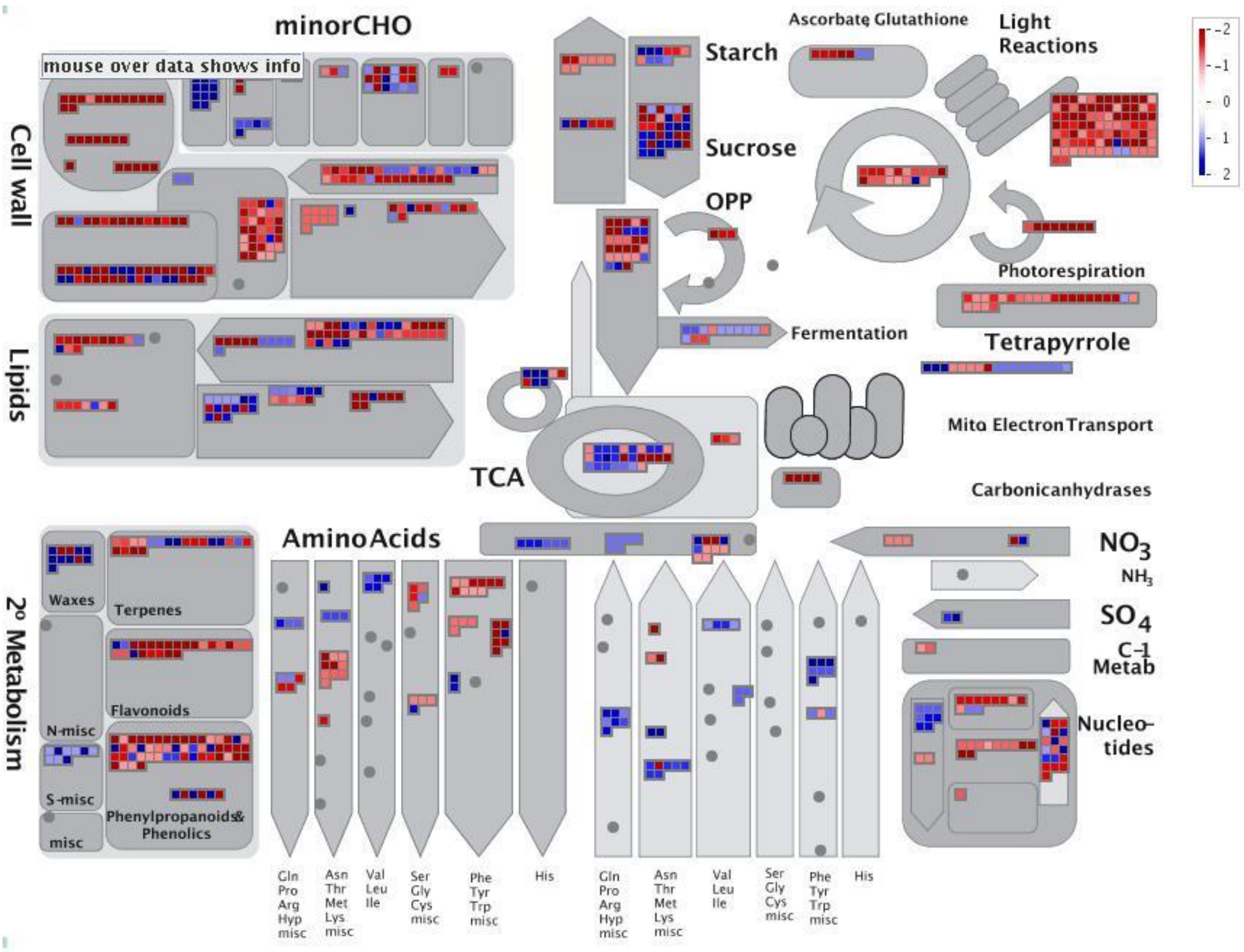
minorCHO
mouse over data shows info
Cell wall
Lipids
2° Metabolism
Waxes
Terpenes
Flavonoids
N-misc
S-misc
misc
Phenylpropanoids&
Phenolics
Starch
Sucrose
OPP
Ascorbate Glutathione
Light
Reactions
Photorespiration
Fermentation
Tetrapyrrole
Mito Electron Transport
Carbonicanhydrases
TCA
AminoAcids
NO3
NH3
SO4
C-1
Metab
Nucleo-
tides
Gln
Pro
Arg
Hyp
misc
Asn
Thr
Met
Lys
misc
Val
Leu
Ile
Ser
Gly
Cys
misc
Phe
Tyr
Trp
misc
His
-2
-1
0
1
2

(C)

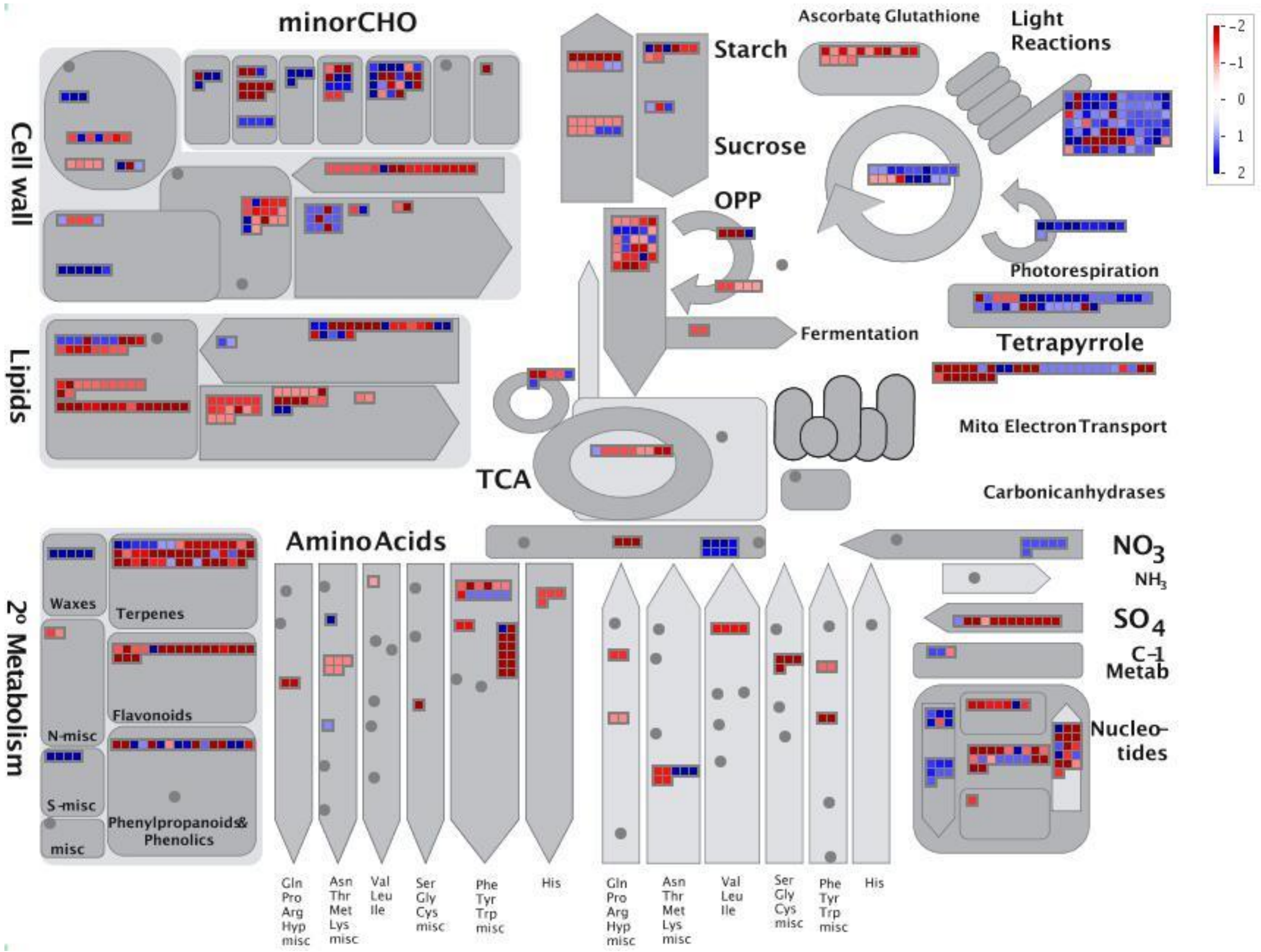
minorCHO
Cell wall
Lipids
Starch
Sucrose
OPP
Ascorbate Glutathione
Light
Reactions
-2
-1
0
1
2
Photorespiration
Fermentation
Tetrapyrrole
Mito Electron Transport
TCA
Carbonicanhydrases
AminoAcids
NO3
NH3
SO4
C-1
Metab
Nucleo-
tides
2° Metabolism
Waxes
Terpenes
Flavonoids
N-misc
S-misc
misc
Phenylpropanoids&
Phenolics
Gln
Pro
Arg
Hyp
misc
Asn
Thr
Met
Lys
misc
Val
Leu
Ile
Ser
Gly
Cys
misc
Phe
Tyr
Trp
misc
His

(D)

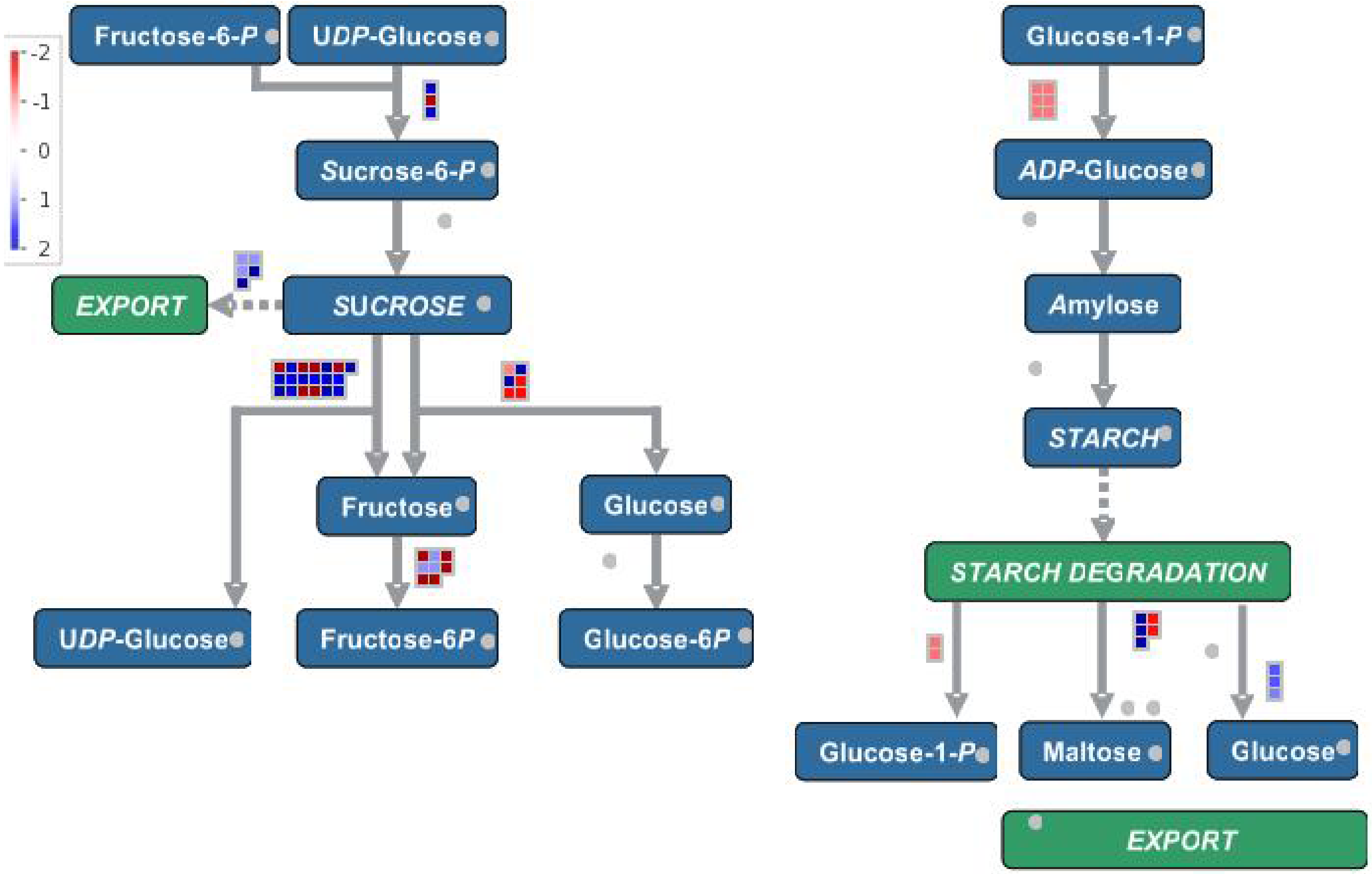

-2
-1
0
1
2
Fructose-6-P
UDP-Glucose
Sucrose-6-P
EXPORT
SUCROSE
Fructose
Glucose
UDP-Glucose
Fructose-6P
Glucose-6P
Glucose-1-P
ADP-Glucose
Amylose
STARCH
STARCH DEGRADATION
Glucose-1-P
Maltose
Glucose
EXPORT

(E)

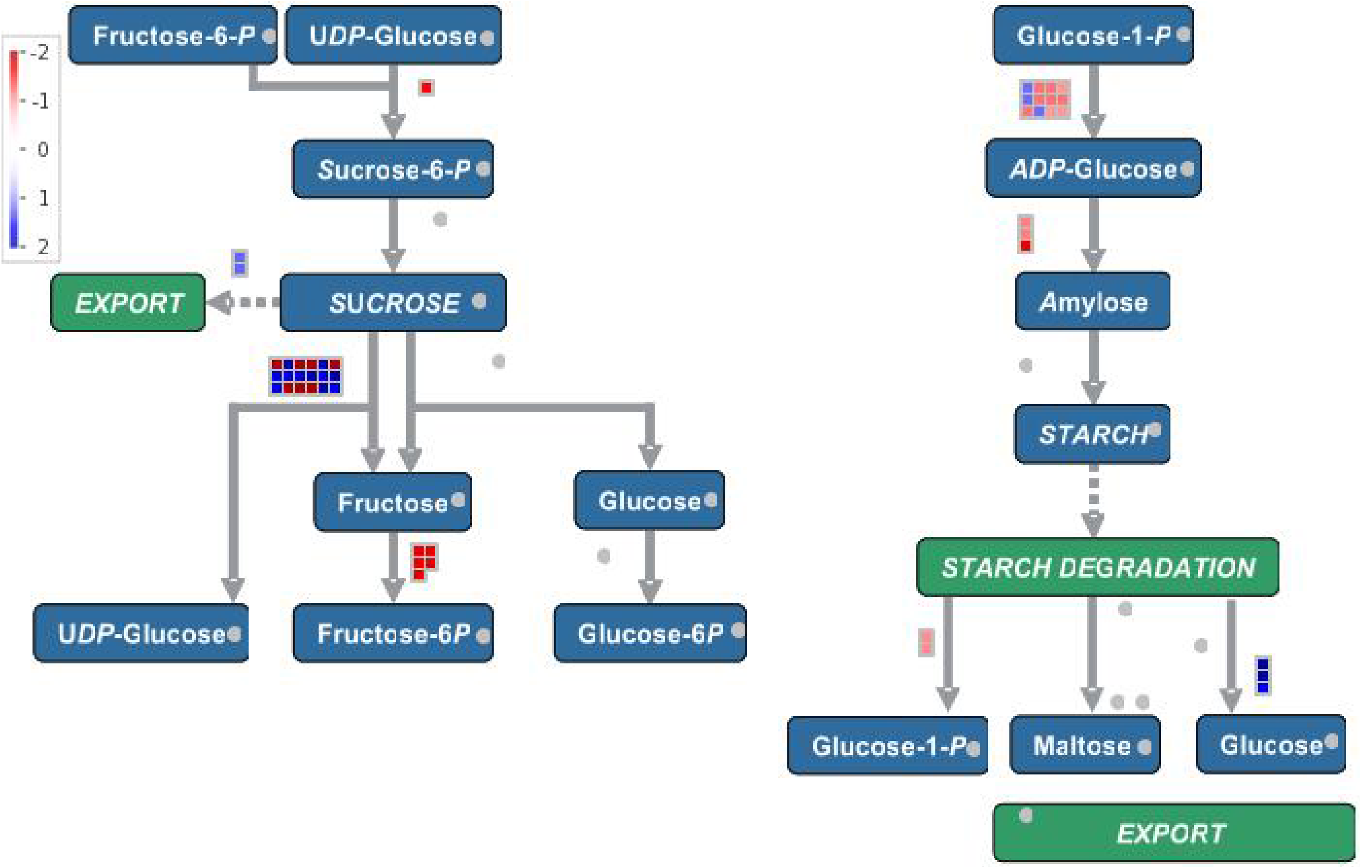
-2
-1
0
1
2
Fructose-6-P
UDP-Glucose
Sucrose-6-P
EXPORT
SUCROSE
Fructose
Glucose
UDP-Glucose
Fructose-6P
Glucose-6P
Glucose-1-P
ADP-Glucose
Amylose
STARCH
STARCH DEGRADATION
Glucose-1-P
Maltose
Glucose
EXPORT

(F)

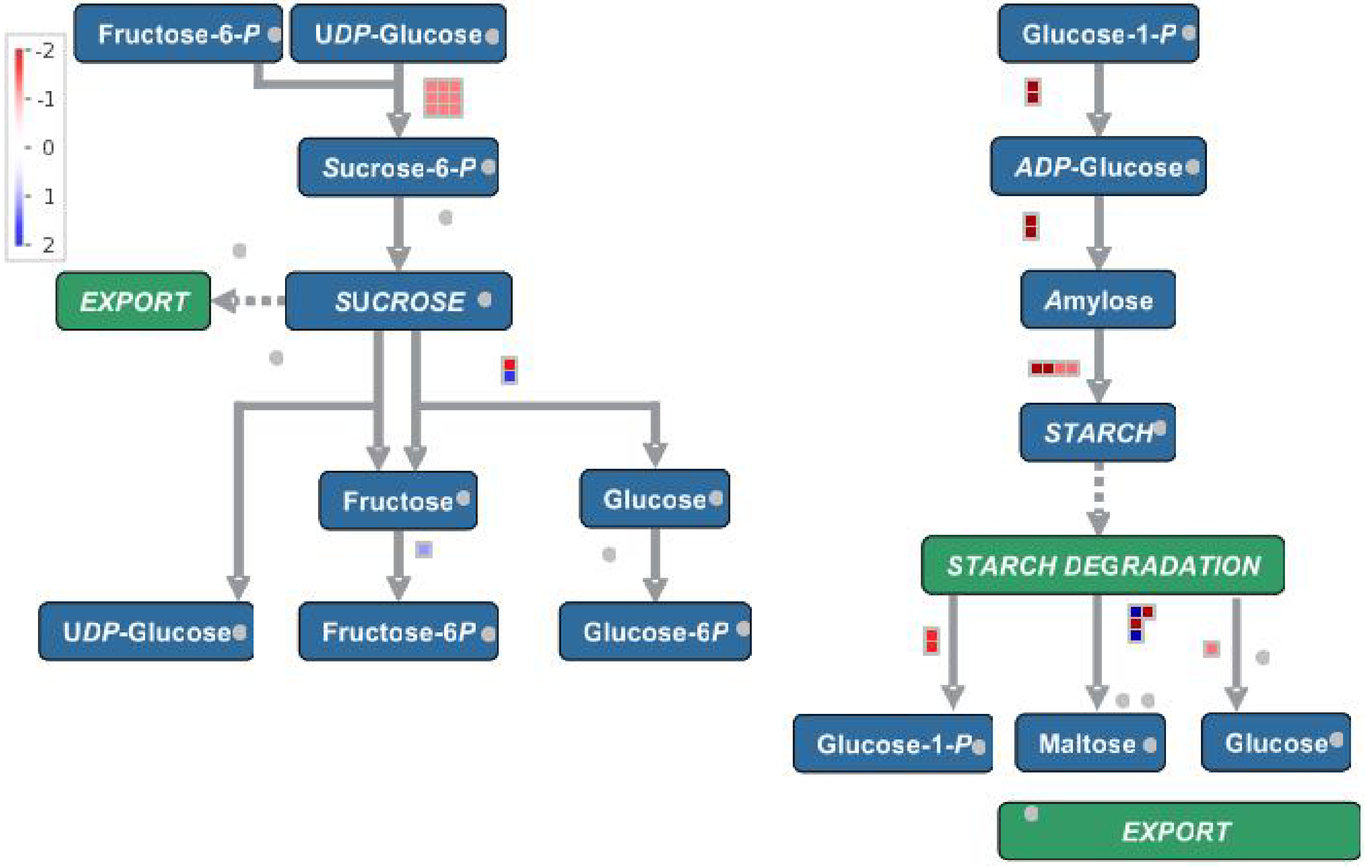

Fructose-6-P
UDP-Glucose
-2
-1
0
1
2
Sucrose-6-P
EXPORT
SUCROSE
Fructose
Glucose
UDP-Glucose
Fructose-6P
Glucose-6P
Glucose-1-P
ADP-Glucose
Amylose
STARCH
STARCH DEGRADATION
Glucose-1-P
Maltose
Glucose
EXPORT

Figure S7 Overview of different metabolic pathways and carbohydrates affected by the differentially expressed genes in response to drought and water recovery treatments. Metabolic pathways associated with differentially expressed genes after the 3-day drought treatment (**A**), 6-day drought treatment (**B**), and water recovery period (**C**). Carbohydrate metabolic pathways associated with differentially expressed genes after the 3-day drought treatment (**D**), 6-day drought treatment (**E**), and water recovery period (**F**). In each panel, the expression levels of up- and down-regulated genes are indicated in blue and red, respectively.

	3d	6d	Re_watered	
GRMZM2G113432	-1.11	-2.37	-0.22	cellulose
GRMZM2G173759	-1.57	-1.27	0.51	
GRMZM2G015886	0.00	-2.32	0.00	
GRMZM2G018241	-0.43	-1.01	-0.32	
GRMZM2G025231	-0.51	-1.45	-0.42	
GRMZM2G027723	-0.97	-1.93	-0.57	
GRMZM2G028353	-0.04	-1.00	-0.45	
GRMZM2G074546	-1.15	-2.17	1.39	
GRMZM2G082580	-0.85	-1.97	-0.22	
GRMZM2G109326	0.33	-1.83	1.06	
GRMZM2G110145	-0.43	-1.29	-0.43	
GRMZM2G112336	-0.30	-1.11	-0.67	
GRMZM2G113137	-0.54	-1.03	-0.65	
GRMZM2G115772	-0.59	-1.52	-0.25	
GRMZM2G142898	-0.84	-1.67	0.31	
GRMZM2G424832	-0.42	-1.39	0.01	
GRMZM5G826714	-0.31	-1.00	-0.77	
GRMZM2G177631	-0.74	-1.67	-1.09	
GRMZM2G010142	-0.50	-0.77	-1.49	
GRMZM2G028286	-0.63	-0.54	-1.44	
GRMZM2G039454	-0.26	0.46	-1.06	
GRMZM2G071970	0.00	-0.78	-1.01	
GRMZM2G074792	-0.14	-0.10	-1.32	
GRMZM5G870176	-0.63	0.17	-1.90	
GRMZM2G122431	1.51	1.58	1.70	
GRMZM2G014558	0.48	0.81	3.23	
GRMZM2G445169	0.00	0.60	0.00	expansin
GRMZM2G176595	-2.76	-3.53	0.16	
GRMZM2G082520	-1.73	-5.69	0.67	
GRMZM2G004955	-0.84	-2.45	2.47	
GRMZM2G013002	-0.69	-1.60	1.06	
GRMZM2G013671	1.12	-2.39	0.00	
GRMZM2G021621	-1.15	-1.85	0.91	
GRMZM2G056236	0.19	-3.95	-0.24	
GRMZM2G327266	0.51	-3.94	0.62	
GRMZM2G339122	-0.15	-3.13	0.53	
GRMZM2G361064	-1.26	-3.46	1.57	
GRMZM2G154178	-1.01	-3.42	-0.73	
GRMZM2G026147	0.68	2.67	0.20	
GRMZM2G095968	2.86	2.87	2.38	
GRMZM5G859316	3.98	2.57	1.26	
GRMZM2G021427	-1.21	0.00	2.40	
GRMZM2G004699	-0.85	-1.95	0.01	xyloglu can
GRMZM2G039919	-1.63	-2.02	1.02	
GRMZM2G063566	0.26	-2.25	1.60	
GRMZM2G119783	-1.01	-4.65	0.08	
GRMZM2G364748	1.55	1.31	1.45	
AC210669.3_FG001	2.75	-0.84	-1.93	

Figure S8 Expression profile of genes involved in cell wall development. Cellulose, expansin and xyloglucan endotransglucosylase related genes are involved in cell wall development. The fold change value (log2(MT/WT)) in the figure is calculated. Green color represent down-regulated genes. Red color represent up-regulated genes.
